# Machine-learned wearable sensors for real-time hand-motion recognition: toward practical applications

**DOI:** 10.1093/nsr/nwad298

**Published:** 2023-11-27

**Authors:** Kyung Rok Pyun, Kangkyu Kwon, Myung Jin Yoo, Kyun Kyu Kim, Dohyeon Gong, Woon-Hong Yeo, Seungyong Han, Seung Hwan Ko

**Affiliations:** Department of Mechanical Engineering, Seoul National University, Seoul08826, South Korea; Department of Mechanical Engineering, Seoul National University, Seoul08826, South Korea; IEN Center for Human-Centric Interfaces and Engineering, Institute for Electronics and Nanotechnology, Georgia Institute of Technology, Atlanta, GA30332, USA; School of Electrical and Computer Engineering, Georgia Institute of Technology, Atlanta, GA30332, USA; Department of Mechanical Engineering, Seoul National University, Seoul08826, South Korea; Department of Chemical Engineering, Stanford University, Stanford, CA94305, USA; Department of Mechanical Engineering, Ajou University, Suwon-si16499, South Korea; IEN Center for Human-Centric Interfaces and Engineering, Institute for Electronics and Nanotechnology, Georgia Institute of Technology, Atlanta, GA30332, USA; George W. Woodruff School of Mechanical Engineering, Georgia Institute of Technology, Atlanta, GA30332, USA; Department of Mechanical Engineering, Ajou University, Suwon-si16499, South Korea; Department of Mechanical Engineering, Seoul National University, Seoul08826, South Korea; Institute of Advanced Machinery and Design (SNU-IAMD), Seoul National University, Seoul08826, South Korea

**Keywords:** wearable sensor, soft electronics, artificial intelligence, machine learning, human–machine interfaces, gesture recognition

## Abstract

Soft electromechanical sensors have led to a new paradigm of electronic devices for novel motion-based wearable applications in our daily lives. However, the vast amount of random and unidentified signals generated by complex body motions has hindered the precise recognition and practical application of this technology. Recent advancements in artificial-intelligence technology have enabled significant strides in extracting features from massive and intricate data sets, thereby presenting a breakthrough in utilizing wearable sensors for practical applications. Beyond traditional machine-learning techniques for classifying simple gestures, advanced machine-learning algorithms have been developed to handle more complex and nuanced motion-based tasks with restricted training data sets. Machine-learning techniques have improved the ability to perceive, and thus machine-learned wearable soft sensors have enabled accurate and rapid human-gesture recognition, providing real-time feedback to users. This forms a crucial component of future wearable electronics, contributing to a robust human–machine interface. In this review, we provide a comprehensive summary covering materials, structures and machine-learning algorithms for hand-gesture recognition and possible practical applications through machine-learned wearable electromechanical sensors.

## Teaser

This review provides a thorough overview of the current research in machine-learned wearable sensors for real-time hand motion recognition, highlighting current challenges and future directions toward practical applications in reality.

## INTRODUCTION

The human hand is a remarkably versatile anatomical structure that plays a crucial role in a diverse range of activities, encompassing the grasping of objects, the control of devices and the completion of a multitude of everyday tasks [1]. Through the assistance of innate motor and sensory feedback systems, humans are able to seamlessly and rapidly execute a broad range of manual tasks with great precision [2]. In particular, proprioception, the ability to sense the position and movement of the body, is essential in allowing humans to estimate the postural configuration of hands in real time, enabling even greater levels of control and manipulation [3,4].

In the field of soft electronics and wearable technology, the emulation of human proprioception is considered the ultimate goal [5]. In recent decades, soft electronics have created new ways for humans and machines to communicate, and have provided natural and intuitive human–machine interfaces by converting mechanical deformation into electrical signals [6–8]. Therefore, soft electronics have been regarded as suitable platforms to mimic human proprioception. However, most early soft sensors have primarily been effective in highly constrained environments, and thus they have usually failed to yield distinctive signals in time-continuous environments while being worn due to unwanted signals and interference from complex and dynamic hand movements.

Nevertheless, real-time signal processing for soft sensors is a fundamental undertaking in the development of hand-motion recognition and its expansion into real-world application. Real-time signal processing enables immediate feedback and response to dynamic changes in hand motion. Furthermore, it ensures that the collected and analyzed information remains current, thereby reducing the potential for errors that may occur with delayed processing. However, to date, even if soft sensors generate meaningful signals for hand motion in constrained scenarios, humans can only assess the motion of the sensor after the signals have been generated. In addition, traditional methods relying on rule-based systems and algorithms introduce latency in processing sensor data to match complex hand motions with predefined rules for each specific gesture. Consequently, the progress of research regarding wearable technology that uses only soft sensors was confined to the laboratory level, hampering its transition to real-world applications.

Recent advances in artificial intelligence (AI) technology have led to a breakthrough in the development of soft sensors capable of mimicking human proprioception, demonstrating their potential in real-time signal processing [9,10]. In particular, machine-learning algorithms have demonstrated their ability to accurately classify signals generated by hand motions with a high degree of freedom, even in highly complex scenarios with large amounts of data [11–13]. Moreover, they are capable of identifying patterned signals even in the vast amount of undefined data and complex behaviors of sensors due to the non-linearity and hysteresis of the sensors, showing potential for soft sensors to be used in unconstrained environments. As a result, the seamless integration of soft sensors and AI technologies has become a crucial issue for achieving rapid and precise hand-motion recognition.

**Figure 1.**
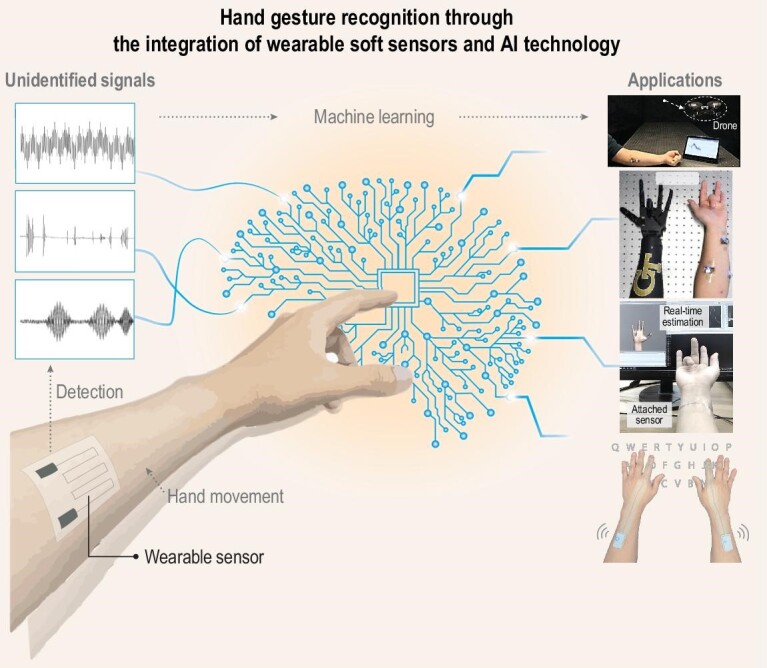
Conceptual illustration of hand-gesture recognition through the integration of wearable soft sensors and AI technology. Reproduced with permission from ref. [18]; copyright 2020 Springer Nature. Reproduced with permission from ref. [58]; copyright 2020 Springer Nature. Reproduced with permission from ref. [21]; copyright 2023 Springer Nature.

Numerous reviews have covered the areas of material and structure design in the fabrication of soft sensors, as well as their potential application [14–16]. Therefore, this review briefly introduces the material and structural strategies employed in developing soft sensors and machine-learning algorithms for data processing and identification. Subsequently, this review mainly focuses on the utilization of AI-assisted soft sensors for real-time hand-motion estimation and their practical applications, such as machine-control systems [17,18], text-input systems [19–21], and augmented-reality (AR) and virtual-reality (VR) applications (Fig. 1) [22,23]. Finally, this review discusses the challenges and outlook of AI-integrated soft sensors with regard to their practical application in real-world scenarios.

## HAND-MOTION ANALYSIS

**Figure 2.**
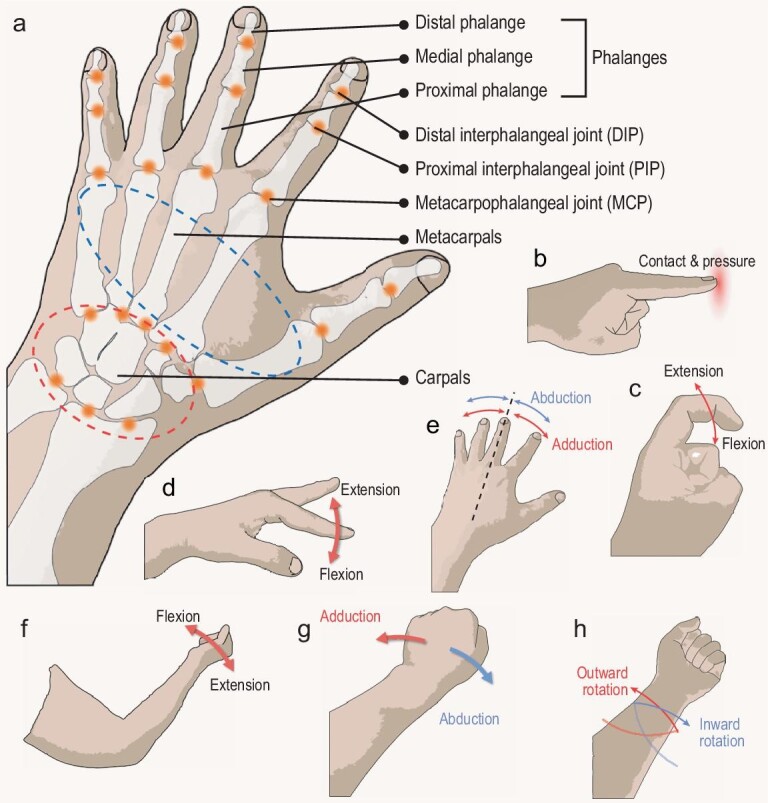
Graphical illustrations for analysis of the anatomy of the human hand and various human hand motions. (a) Anatomy of the human hand. (b) Contact and pressure at the finger. (c) Flexion and extension of the interphalangeal joints of the finger. (d) Flexion and extension and (e) abduction and adduction of the metacarpophalangeal joints of the finger. (f) Flexion and extension and (g) abduction and adduction of the wrist. (h) Inward and outward rotation of the wrist.

The human hand stands out as an exceptionally flexible and versatile organ, owing to its distinctive anatomical features and capabilities [24]. Its intricate structure, encompassing multiple bones, joints, tendons and muscles, facilitates a broad range of motion and dexterity. The positioning of bones, including the carpals, metacarpals and phalanges, along with the presence of joints like the distal interphalangeal joints (DIPs), proximal interphalangeal joints (PIPs) and metacarpophalangeal joints (MCPs) within the hand, allows for intricate movements and precise manipulation of objects (Fig. 2a). Moreover, the complex network of muscles and tendons of the hand enables coordinated and precise finger movements, essential for delicate tasks.

Figure 2 also provides basic examples of real-life hand motions made possible by the fingers and wrists, which allow for a wide range of actions and interactions with the environment. Firstly, contact and pressure encompass tactile and forceful interactions between the hand and objects or surfaces, playing a crucial role in how human beings perceive and manipulate the physical world (Fig. 2b). In addition, for fingers, the interphalangeal joints including DIPs and PIPs permit rotational movements along the coronal axis, encompassing flexion and extension (Fig. 2c). Moreover, the MCPs in the fingers enable bending along both the coronal and sagittal axes, with their movements commonly referred to as flexion and extension (Fig. 2d) and abduction and adduction (Fig. 2e). The wrist significantly broadens the range of hand movements. It can rotate around both the coronal and sagittal axes, commonly referred to as flexion and extension (Fig. 2f) and abduction and adduction (Fig. 2g). Additionally, the wrist possesses the ability to twist along the vertical axis, a motion known as inward rotation and outward rotation (Fig. 2h). Lastly, considering the dexterity of the human hand and the number of fingers it possesses, a multitude of hand gestures can be executed, stemming from the basic gestures above and their combinations. Despite the numerous and intricate hand gestures that the human hand can perform, humans possess the remarkable ability to instinctively and accurately perceive these hand motions thanks to the complex network of nerves connecting the hand to the brain. In the pursuit of developing various applications for hand-motion recognition, it is essential to replicate the cognitive capabilities of humans when identifying hand gestures.

## MATERIAL AND STRUCTURE DESIGN FOR WEARABLE MECHANICAL SENSORS

To replicate the functionalities of human sensory systems, numerous wearable sensors are currently being developed and reported in the scientific literature, and are designed to both monitor a wide range of hand motions and gather associated biomechanical signals from human bodies. When embarking on creating these innovative devices, selecting construction materials is a primary consideration for researchers [25]. Such materials must possess the capacity to withstand the mechanical deformations associated with human motion, including bending, tension and torsion, while still performing stably. Simultaneously, these materials must be capable of accurately measuring an array of biomechanical and physiological signals, produced by the skin and muscles in response to tension and compression forces inherent in body movements. For this reason, materials selected for wearable sensor construction often demonstrate superior elasticity. In addition, when the use of rigid materials is inevitable, they are fabricated to be thin and integrated with various structural designs and patterns, which endow them with requisite flexibility and stretchability. This section offers a summary of the commonly used materials in wearable sensors specifically developed for monitoring human hand movements. It also presents an introduction to the design strategies employed to imbue these materials with flexibility and stretchability. Furthermore, it summarizes the mechanisms by which these sensors measure biomechanical signals and the specific target signals they are designed to capture from the human body.

### Material choices for wearable mechanical sensors

**Figure 3.**
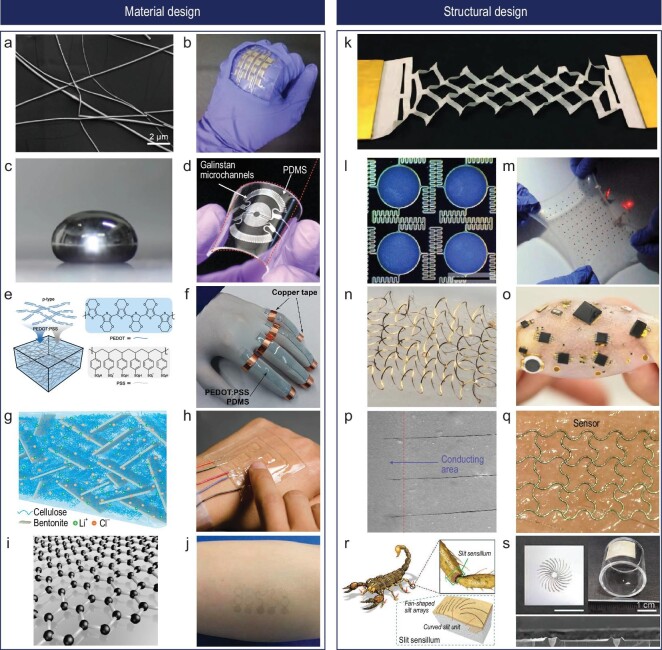
Various designs of materials and structures for wearable sensors that monitor hand movement. (a–j) Sensor constituent materials. (a) Scanning electron microscope (SEM) image of the Ag NWs percolation network (scale bar: 2 μm). Reproduced with permission from ref. [26]; copyright 2017 American Chemical Society. (b) Photograph of the Ag NW-based multi-axial strain sensor on a human hand. Reproduced with permission from ref. [29]; copyright 2015 American Chemical Society. (c) Digital image of the liquid metal droplet. Reproduced with permission from ref. [32]; copyright 2021 American Chemical Society. (d) Optical image of the liquid metal-based pressure sensor. Reproduced with permission from ref. [34]; copyright 2017 John Wiley and Sons. (e) Schematic image of PEDOT: polystyrene sulfonate (PSS) conductive polymer and its molecular structures. Reproduced with permission from ref. [37]; copyright 2021 Springer Nature. (f) Photograph of PEDOT: PSS-based strain sensors on fingers. Reproduced with permission from ref. [40]; copyright 2015 John Wiley and Sons. (g) Schematic illustration of the structure of the hydrogel-based conductive polymer. Reproduced with permission from ref. [41]; copyright 2022 Springer Nature. (h) Digital image of an ionic hydrogel-based strain sensor on a human finger. Reproduced with permission from ref. [46]; copyright 2014 John Wiley and Sons. (i) Schematic illustration of a hexagonal lattice of graphene. Reproduced with permission from ref. [184]; copyright 2017 Elsevier. (j) Optical image of the graphene-based tattoo-like sensor on human skin. Reproduced with permission from ref. [49]; copyright 2017 American Chemical Society. (k-s) Designing structural elasticity: (k) Kirigami structure employed to enhance the stretchability of graphene thin film. Reproduced with permission from ref. [51]; copyright 2015 Springer Nature. (l) Optical image of serpentine structures to boost the flexibility of otherwise rigid materials. Reproduced with permission from ref. [54]; copyright 2013 Springer Nature. (m) Digital image of the stretchable battery under biaxial strain. Reproduced with permission from ref. [54]; copyright 2013 Springer Nature. (n) Angled optical image of helical coil structures aimed at improving flexibility. Reproduced with permission from ref. [56]; copyright 2017 Springer Nature. (o) Photograph of a stretchable device with rigid components enabled by the helical coil structures. Reproduced with permission from ref. [56]; copyright 2017 Springer Nature. (p) SEM image of the laser-induced crack on the Ag NPs layer. Reproduced with permission from ref. [58]; copyright 2020 Springer Nature. (q) Digital image of the micropatterned crack sensor on skin. Reproduced with permission from ref. [58]; copyright 2020 Springer Nature. (r) Graphical illustrations of the bionic structure of a slit sensillum on the working leg of a scorpion. Reproduced with permission from ref. [61]; copyright 2022 John Wiley and Sons. (s) Photograph of the sensor with scorpion-inspired bionic structure (top) and SEM image showing a cross-sectional view of the structure (bottom). Reproduced with permission from ref. [61]; copyright 2022 John Wiley and Sons.

The field of wearable sensors for hand-motion detection is evolving rapidly, with a frequent emphasis on materials known for their softness, flexibility and stretchability. These typically include metal nanowires (NWs), liquid metal and conductive polymers. In particular, one-dimensional metal NWs have the unique feature of forming random network structures at the nanoscale (Fig. 3a) [26]. This feature, combined with percolation theory, allows for detailed control and understanding of the overall conductivity of the material [27]. Owing to their flexibility and stretchability, these NWs can be fabricated into thin films with thread-like mechanical properties, making them an ideal choice for components such as electrodes and sensitive elements within wearable sensors (Fig. 3b) [28,29]. Among the different types of NWs, silver (Ag) and copper (Cu) NWs stand out. These can be swiftly synthesized at room temperature via solution-based processes, thus giving them relatively low production costs. Also, Ag NWs demonstrate exceptional reproducibility and offer users the flexibility to adjust the aspect ratio of the NW length by fine-tuning the synthesis parameters, accommodating the specific requirements of their applications [30].

Liquid metal, another noteworthy material, remains in a liquid state at room temperature due to its low melting point, meaning it exhibits nearly limitless flexibility and stretchability (Fig. 3c) [31,32]. Gallium, a prominent representative of liquid metals, is commonly employed due to its outstanding biocompatibility (Fig. 3d) [33,34]. When compared to NWs, liquid metals show high conductivity and superior resilience to deformation, thereby fitting the bill for repetitive use across a broad range of human movements. Liquid metals are typically not used alone; instead, they are embedded within soft silicone-based polymers like polydimethylsiloxane (PDMS) or Ecoflex to craft various wearable sensors [35]. Nevertheless, liquid metals do come with certain challenges. They are susceptible to oxidation when exposed to oxygen in the air, necessitating steps to overcome this chemical instability. Furthermore, biocompatibility validation is required for metals other than gallium. The fabrication process is relatively intricate and demands high precision, contributing to increased production costs and making mass production more challenging.

Organic polymer composites, assembled through simple electrical polymerization processes, have also shown promise in the realm of wearable sensors [36]. Conductive polymers are typically distinguished by the type of bonded charge they possess, such as π-bonding or ions. Among these, conjugated polymers, containing π-electron bonds within their backbone chains, are drawing significant attention (Fig. 3e) [37]. They can be fabricated much more cost effectively than their inorganic counterparts while offering a variety of benefits including mechanical flexibility and impact resistance [38]. Conjugated polymers include notable substances such as polyacetylene (PA), polythiophene (PT), poly(3,4-ethylenedioxythiophene) (PEDOT), polypyrrole (PPy), polyphenylene and polyaniline (PANi). PEDOT-based polymers, in particular, have demonstrated their applicability as wearable sensors due to their high conductivity, elasticity and durability, allowing for usage even amidst changes in curved shapes (Fig. 3f) [39,40].

In addition, conductive hydrogels stand out among conductive polymers (Fig. 3g) [41]. Hydrogel is a material composed of a three-dimensional network of hydrophilic polymer chains that are highly swollen with water or other aqueous solutions. It creates a gel-like substance with properties akin to natural tissues, providing seamless and comfortable wearability due to its very low mechanical mismatch with human skin [42]. However, in its natural state, pure hydrogel is non-conductive. Enhancement of electrical conductivity can be achieved by introducing conductive materials into the hydrogel matrix or through chemical modification, such as the incorporation of conductive groups or functionalization. Like other conductive materials, conductive hydrogel produces electrical signals as a result of alterations in polymer chain spacing and ion concentration in various regions of the gel during mechanical deformation. Owing to their high moisture content and biocompatibility, they have proven themselves as viable materials for wearable sensors [43]. Based on the Flory–Rehner theory, hydrogels can be tailored to user requirements by adjusting parameters such as polymer volume fraction, polymer chain molecular weight and mesh size during the polymerization process. Such hydrogel-based wearable sensors have proven their ability to reliably and linearly detect human movement, with high transparency and elasticity (Fig. 3h) [44–46].

Lastly, two-dimensional (2D) materials like graphene [47] and MXene [48] are promising candidates for wearable electromechanical sensors (Fig. 3i). They are inherently ultrathin and flexible, allowing them to conform to the skin surface and the deformation of skin even after being fabricated as sensors (Fig. 3j) [49]. Furthermore, 2D materials are highly conductive because of their atomic structure, which consists of a single layer of atoms arranged in a lattice [50]. This structure minimizes charge carrier scattering, provides a large surface area for electron transport, and allows for unique quantum effects, resulting in efficient electron movement. However, the production of high-quality 2D materials is still challenging and expensive, limiting their scalability for large-scale sensor manufacturing. Moreover, designing 2D materials as sensors requires specialized fabrication techniques incompatible with other fabrication processes, which can limit their practicality in specific applications. Achieving seamless contact between the 2D material and other components can be challenging due to their atomic thickness, affecting device performance.

Numerous conducting materials such as metal-based materials, carbon-based materials and transition metal dichalcogenides (TMDCs) are also suitable materials for wearable electromechanical sensors [16]. Furthermore, recent research has been actively exploring the use of conducting materials in composite forms to fabricate various wearable sensors. Therefore, selecting and designing materials appropriately is a crucial factor in fabricating desired sensors and achieving their high performance.

### Structural elasticity in wearable mechanical sensor design

When creating flexible and stretchable wearable sensors, it is common to engineer flexibility into rigid materials via structural design. This often proves more effective than relying on the inherent properties of the materials alone. For example, techniques inspired by traditional origami or kirigami have been leveraged to infuse stretchability into otherwise rigid films and nanomaterials (Fig. 3k) [51]. This approach has enabled the production of sensors that can withstand strains ranging from 100% to 400%, proving particularly useful in wearable devices designed to monitor human motion [52,53].

Serpentine structures offer another effective strategy for enhancing the flexibility of rigid materials (Fig. 3l) [54]. By adopting wave-like serpentine patterns, even metal films with minimal flexibility can be transformed into wearable sensors that can sustain strains up to 250% (Fig. 3m) [55]. There have also been accounts of utilizing coil-like three-dimensional (3D) structures (Fig. 3n), as opposed to wave-like ones, to further increase material flexibility and optimize interaction with skin (Fig. 3o) [56]. The engineering of macro-scale designs is not the only way to introduce stretchability. Repetitive mesh structures on the nanometer-to-millimeter scale can also be employed to this end [57]. Nevertheless, while these various structural design techniques improve mechanical performance, they typically result in a decrease in sensor sensitivity. To offset this, research has been conducted where micro-cracks are intentionally induced on the patterned metallic films (Fig. 3p) [58,59]. This not only maintains material flexibility but also significantly enhances sensor performance (Fig. 3q).

Another important design approach for wearable sensors is a bionic structure that uses the insight researches have gained into biological systems or organisms to mimic certain aspects of living organisms to improve their performance in specific applications [60]. Many organisms in nature possess remarkable sensing abilities, and mimicking these sensing capabilities has been proven to be of tremendous assistance in enhancing performance. As an example, a bio-inspired supersensitive strain sensor that mimics the working legs of scorpions was fabricated (Fig. 3r) [61]. Scorpions have evolved a remarkably powerful vibration sensory organ on their walking legs known as a ‘slit sensillum’. This sensory organ enables them to detect subtle vibrations and remain active predators, even when their vision is lost. The key to this sensory ability lies in the curved slits and their fan-shaped arrangement on the slit sensillum, allowing scorpions to sense vibrations from any direction with high sensitivity. By replicating the structure of the walking legs of the scorpion, the authors have created an omnidirectional and highly sensitive strain sensor capable of detecting vibrations (Fig. 3s). The concept of a bionic structure can inspire a highly effective strategy for enhancing a capability of the sensor to perceive and process information. This approach encompasses natural entities, including animals, plants and even humans. Recently, sensors have emerged that not only mimic structures but also replicate signal-processing methods found in humans [62].

### Device configurations and sensing mechanisms of wearable mechanical sensors

Wearable soft sensors, especially electromechanical sensors, are designed to convert mechanical deformations caused by hand movements into electrical signals. They can be categorized into various types of sensors that employ different mechanisms to detect mechanical deformation. Prominent among these are the piezoresistive, capacitive, piezoelectric, triboelectric and field-effect transistor (FET) type sensors [63].

**Figure 4.**
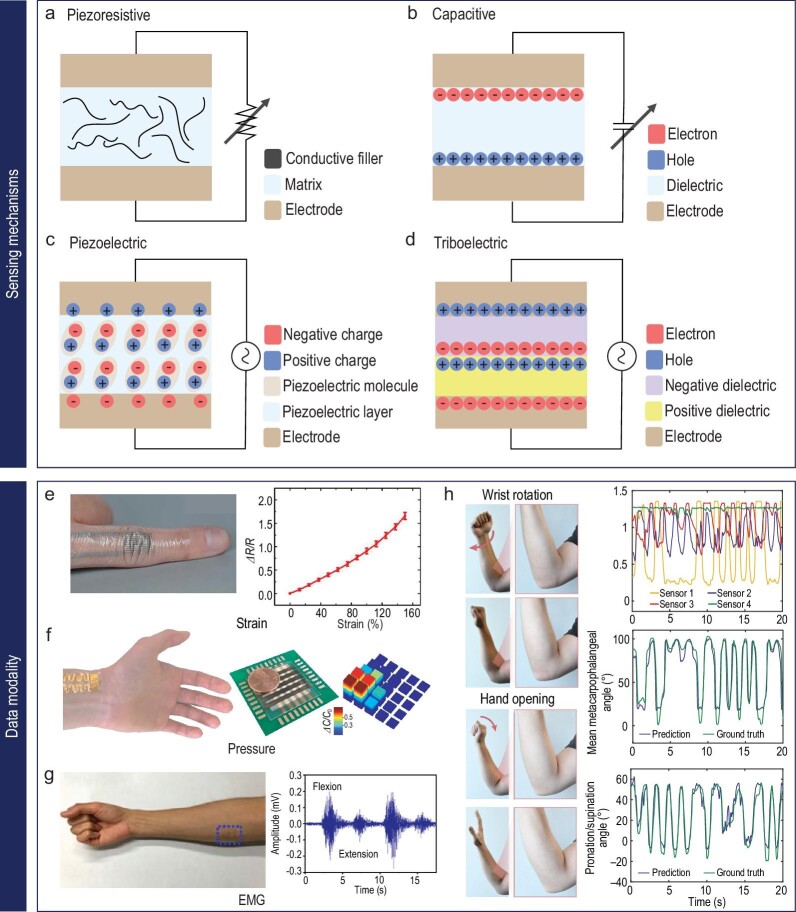
Mechanisms and signals related to hand motion. Schematic representation of (a) piezoelectric, (b) capacitive, (c) piezoelectric and (d) triboelectric sensing mechanisms. Identifying target signals for hand-motion recognition: (e) strain sensor used for hand-motion detection. Reproduced with permission from ref. [78]; copyright 2021 American Association for the Advancement of Science. (f) Configuration of multiple sensors for pressure detection. Reproduced with permission from ref. [82]; copyright 2020 Springer Nature. (g) EMG signals corresponding to hand motion. Reproduced with permission from ref. [85]; copyright 2019 American Chemical Society. (h) Intricate sensor signals due to multiple sensors and complex movement of motion hand. Reproduced with permission from ref. [79]; copyright 2014 Springer Nature.

Piezoresistive sensors function through the piezoresistive effect, where the electrical resistance of the material alters with mechanical deformation (Fig. 4a). When piezoresistive materials experience strain or stress, the arrangement or density of fillers within them changes. Consequently, the resistance of the piezoelectric material usually varies in proportion to the applied mechanical deformation. This change in resistance can be measured and correlated with the applied strain or pressure. The ability to directly convert mechanical deformation into electrical signals makes this mechanism highly practical. Depending on the sensor design, it can show high sensitivity, making it well-suited for measuring subtle signals like breathing and heartbeat [64,65]. However, the output of sensor can be affected by variations in external temperature, necessitating sensor calibration and compensation [66].

A capacitive electromechanical soft sensor is a sensor that employs variations in capacitance for detecting mechanical deformations in soft materials. Capacitance refers to the ability of conductors and insulators to store electric charge. This sensor consists of two conductive layers separated by a dielectric (insulating) layer, effectively forming a capacitor (Fig. 4b). The system should include a circuit to gauge the capacitance of the sensor. When the soft material undergoes deformation, the capacitance changes, leading to modifications in the electrical signals measured by the circuit. These alterations in capacitance are then processed and analyzed to provide insight into the mechanical state or deformations of the soft material [67]. Despite their high sensitivity, resolution and non-contact characteristics, they may cause unforeseen capacitance alterations if other objects with different dielectric constants are present near the sensor [68].

Piezoelectric and triboelectric-type mechanical sensors employ similar mechanisms for the generation of electrical voltage in response to mechanical deformation. The piezoelectric mechanism is designed to use the charges or voltage generated on the crystal planes of a material due to mechanical deformation (Fig. 4c) [69]. When strain or pressure is applied to a piezoelectric material, its internal crystal structure gets distorted, causing a separation of positive and negative charges. This leads to the development of an electric potential across the material, creating an electrical signal.

Triboelectric-type sensors hinge on the triboelectric effect, which produces voltage through contact, separation, or friction between two different materials (Fig. 4d) [70,71]. The triboelectric effect occurs when certain materials come into contact and then separate, causing the transfer of electric charges between them. When two different materials come into contact and slide against each other, one material acquires a positive charge as it loses electrons, while the other material obtains a negative charge by gaining electrons. This charge separation results in an electric potential difference between the materials, leading to the generation of an electrical signal.

Harnessing piezoelectric and triboelectric mechanisms, mechanical sensors based on piezoelectric nanogenerators (PENGs) and triboelectric nanogenerators (TENGs) have been developed for health monitoring sensors [72]. These sensors use the energy generated by human movement as their power source, offering advantages such as simplicity, cost-effectiveness, relatively high sensitivity and self-powering capabilities. However, sensor efficiency depends on the use of materials with distinct electron affinities, which can make them sensitive to external factors like humidity and temperature. While their performance may be influenced by these environmental conditions, it is important to note that these sensors are designed to excel in detecting subtle mechanical signals. Their sensitivity enables them to capture fine biomechanical signals resulting from activities like muscle contractions or breathing. By carefully optimizing their materials and design, we can strike a balance between sensitivity and robustness, ensuring reliable performance even in varying environmental conditions. When combined with composite strips, electrodes incorporating the piezoelectric effect are used as sensors to detect deformations at sites of non-linear movements, such as the jaw, arms and knees [73].

FET sensors have played a significant role in the device configuration of wearable soft sensors [74,75]. FETs are essential components in integrated circuits, which typically comprise a semiconductor channel connecting two electrical terminals, referred to as the source and drain, along with a third terminal known as the gate. As a mechanical sensor, FETs function by detecting changes in the electrical conductance of the semiconductor channel when subjected to mechanical stimuli. In the context of soft mechanical FET sensors, the selection and placement of sensing materials become critical, as they significantly determine the sensing mechanisms, the device structure and sensor performance. In general, FET-based soft mechanical sensors exhibit higher sensitivity, enabling them to detect even subtle mechanical deformations [76,77]. An intriguing aspect is that FET sensors can be scaled down to the micro-nanoscale, facilitating the development of miniature soft sensors ideal for embedding in confined spaces and creating sensor arrays, thereby making them suitable for a range of multimodal sensing applications. Furthermore, FET sensors seamlessly integrate with electronic-readout circuits and signal-processing components, streamlining the processes of data acquisition and analysis. These characteristics position FET-based devices as powerful tools in sensing applications, despite involving slightly more intricate manufacturing processes and additional electronics.

### Target signals for hand-motion recognition

Wearable sensors designed to monitor hand movements primarily function by detecting skin deformations induced by these movements, or by observing action potentials that stem from muscle nerve activation. During actions such as bending or clenching of the hand, which prompt mechanical changes in the skin, these sensors gauge the strain experienced by the skin (Fig. 4e) [78]. To improve the reliability and precision of the signals collected, it is critical to ensure a conformal contact between the sensor and the skin. To facilitate this conformal contact on the wrinkled surface of the skin, sensors are typically fashioned from soft materials and are progressively designed to be thinner. Because strain wearable sensors necessitate the direct measurement of skin deformation, they tend to favor the piezoresistive method [45]. This approach can boost sensitivity, based on the sensor design, enabling the detection of signals from areas of minimal deformation such as the back of the hand or the arm, thereby playing a critical role in precisely identifying hand movements [79].

Beyond hand movements, wearable pressure sensors have been developed to register the pressure that arises when handling or making contact with specific objects [80,81]. Although wearable sensors deployed for strain measurement are generally considered capable of also detecting pressure, understanding the direction of the force imparted onto the skin by external elements, as well as the distribution ratio of this force in various directions, can pose challenges. Consequently, numerous capacitive sensor designs have been reported, each developed to independently measure the force in each direction. As an example of these sensors, a capacitive and transistor-based pressure sensor was developed, which can discern the shape of an object or perceive texture by emulating fingerprints, thanks to the arrangement of multiple sensors that can pinpoint the source of the pressure (Fig. 4f) [80,82–84].

Another pivotal signal closely related to hand motion is the electromyography (EMG) signal. Hand movements are instigated by muscle activation, which can be identified through the electrical neural signals generated in the muscles (Fig. 4g) [85]. The EMG signal is a favored method due to its relative stability and lesser susceptibility to external influences.

Wearable soft sensors with aforementioned signals such as strain, pressure and EMG have demonstrated remarkable reliability and high performance for basic movements in various highly constrained situations. However, analyzing complex movements becomes challenging due to the increased number of sensors and the higher complexity of the data, making accurate prediction of movements difficult (Fig. 4h) [79]. In addition, the analysis method for the signal may vary depending on the placement of the electrode, underscoring the importance of signal calibration when differentiating between hand movements [86]. Furthermore, these methods grapple with issues such as the inherent variability in human movements, interference from other muscle activities, and the difficulty of detecting subtle movements. Consequently, in parallel with the advancements in materials and structures, AI technologies must be adapted for learning complex patterns in signal data and making accurate predictions, thereby enhancing the accuracy and robustness of hand-motion recognition [87,88].

## MACHINE-LEARNING ALGORITHMS FOR GESTURE RECOGNITION

As human–computer interaction continues to evolve, motion recognition has emerged as a crucial research area, allowing for more intuitive and natural interaction with digital systems. Hand motions, ranging from simple movements to complex sequences, provide a rich modality for non-verbal communication. However, the successful interpretation and detection of these gestures pose significant challenges, owing to the dynamic and high-dimensional nature of the data. An effective approach to tackling these challenges has been the application of machine-learning algorithms that can learn from data and make accurate predictions.

Machine learning, a subset of AI, provides a framework for training models to recognize patterns, make decisions and predict outcomes based on data. Over the years, various machine-learning techniques have been tailored and applied to hand-motion recognition tasks. Traditional machine-learning methods, including supervised and unsupervised learning techniques, have been used to recognize predefined motions or to discover inherent patterns in the data.

More recently, deep-learning techniques have come to the fore for their capacity to model complex, high-dimensional data and capture temporal dependencies, making them particularly suitable for hand-motion recognition. Furthermore, advanced learning strategies such as transfer learning and adaptive learning are being employed to enhance model performance and accommodate the variability in individual user styles and changes over time. The following sections delve into these machine-learning algorithms, their application in hand-motion recognition and recent advancements in the field.

### Traditional machine-learning algorithms

**Figure 5.**
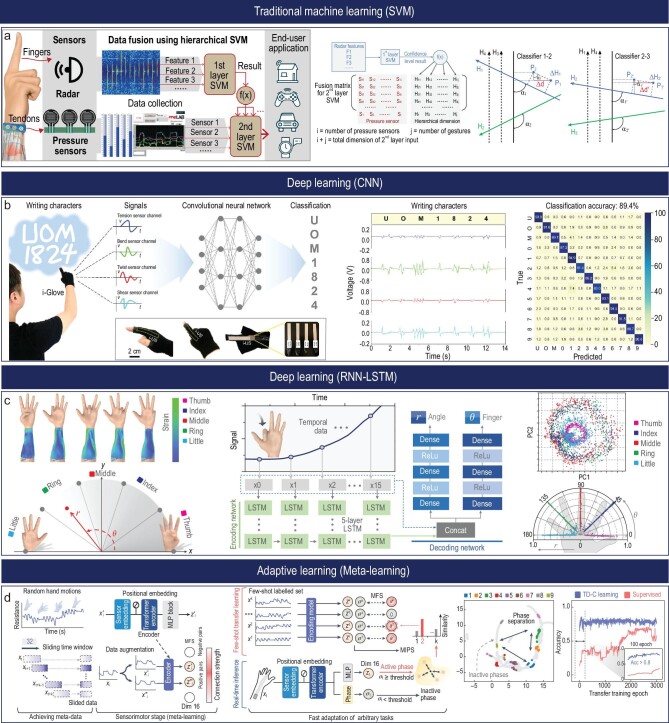
Machine-learning techniques for human hand-posture perception. (a) Schematic diagram of the real-time gesture-recognition system using data fusion with hierarchical SVM. Reproduced with permission from ref. [97]; copyright 2019 John Wiley and Sons. (b) Finger writing character classification with LeNet-5-based CNN network. Reproduced with permission from ref. [106]; copyright 2022 Springer Nature. (c) Schematic diagram of the real-time human–machine interaction application using an LSTM network. Reproduced with permission from ref. [58]; copyright 2020 Springer Nature. (d) Schematic diagram of rapid hand task recognition with meta-learning. Reproduced with permission from ref. [21]; copyright 2023 Springer Nature.

Supervised learning techniques are characterized by their ability to learn a function that maps input data (features) to the desired output, based on a data set where the correct outcomes (labels) are known. These methods include diverse algorithms each with its strengths. For instance, support vector machines (SVMs) are powerful classifiers that work by finding the optimal hyperplane that maximally separates different classes in the feature space. The principal advantage of an SVM lies in its capacity to handle both linear and non-linear data. The flexibility offered by SVMs comes from the choice of kernels, which can transform the data into higher dimensions, making non-linearly separable data linearly separable in the new space. This capacity ensures robust performance in various scenarios, even when the decision boundary between classes is intricate and non-linear [89]. Furthermore, the ‘maximum margin’ approach of SVMs ensures excellent generalization to unseen data, thereby reducing the risk of overfitting, especially when compared to other algorithms that might fit too closely to the noise in the training data. As an example, an SVM has been successfully applied to hand-gesture recognition using surface EMG (sEMG) signals, with researchers achieving high accuracy rates of 99.37% on the training set and 90.33% on the test set [90], and 89.0% in another study [91]. However, while SVMs excel at maximizing the margin of separation, they can sometimes be sensitive to the choice of kernel and hyperparameters. Also, in the case of large data sets or many features, SVMs might not be the most computationally efficient choice. Similarly, the k-nearest neighbors (kNN) technique classifies new instances by measuring similarity to existing instances, with new instances assigned the label of the majority of its ‘k’ nearest neighbors from the training set [92]. One of the primary advantages of kNN is that the algorithm does not make any assumptions about the underlying distribution of data, making it flexible and adaptable to various scenarios. It can be especially effective when the decision boundaries are irregular and not easily captured by linear models. An application utilized kNN in a framework that developed a real-time interactive system capable of recognizing 10 hand gestures as control commands for household appliances [93]. On the downside, kNN can become computationally intensive due to the need to compute distances for every point, and it may also require significant storage to maintain the data set. Moreover, it does not inherently handle data imbalances well. Furthermore, Random Forests constructs a ‘forest’ of decision trees during training, each tree built using a random subset of the data. When predicting the class of a new instance, each tree in the forest casts a vote, and the class with the most votes becomes the prediction of the model [94]. Random Forests are often praised for their ability to handle large data sets with higher dimensionality. A study made use of a Random Forest algorithm-based model for sEMG signal processing and hand-movement recognition, efficiently classifying four types of actions and exhibiting a fast processing speed, thus providing a promising approach for hand-gesture recognition and myoelectric prosthesis control [95]. However, they might not perform as well when data structures are more complex, such as with multi-modal distributions or when relationships between features are more intricate. In the context of hand-gesture recognition, the advantage of supervised learning becomes evident when considering tasks like recognizing a fixed set of gestures [96]. For instance, the innovative application of the hierarchical support vector machine (HSVM) algorithm effectively fuses data from contactless radar and wearable pressure sensors, improving gesture detection accuracy significantly. The combined use of the two sensors via the HSVM algorithm achieved a classification accuracy of 92.5%, outperforming either sensor used independently and even maintaining over 90% accuracy when the pressure sensor data fell below 75% accuracy. This integration showcased the potential of HSVM for data integration in multi-sensor intelligent systems (Fig. 5a) [97]. However, the challenges of supervised learning should also be noted. While they can achieve high accuracy given sufficient labeled data, the need for labor-intensive and time-consuming labeling can be daunting. Moreover, when trying to detect rare or nuanced gestures without sufficient labeled examples, these techniques might struggle. Overfitting can arise if the training data are not representative of real-world scenarios or if models are not adequately regularized [98]. Despite these potential pitfalls, supervised learning techniques remain a cornerstone in the domain due to their generally high performance and interpretability.

Transitioning to unsupervised learning techniques, these algorithms strive to find patterns or structures in the data without relying on provided labels. Clustering algorithms group similar instances together based on their proximity in the feature space. A popular clustering method is the K-means algorithm, which assigns instances to clusters based on their distance to the current cluster center, and then recalibrates the cluster center based on the mean of its instances. One study successfully applied unsupervised clustering algorithms along with supervised methods on two-channel EMG recordings, leading to high classification accuracy in detecting six different hand movements, with the clustering results indicating similar groupings of correctly and incorrectly classified movements [99]. The primary advantage of K-means is its simplicity and ability to scale to large data sets. However, its performance can be sensitive to the initial placement of cluster centroids and might converge to local optima. It is also imperative to specify the number of clusters *a priori*, which can be challenging without domain knowledge [100]. Principal component analysis (PCA), a dimensionality reduction technique, identifies a hyperplane that lies closest to the data and projects the data onto it. The axes on this hyperplane, known as principal components, are specifically chosen to capture the maximum variance in the data [101]. The main advantage is its ability to reduce dimensionality, condensing information into fewer features, which can be beneficial in reducing the computation time and noise in the data. On the downside, while PCA captures the most variance, it might not always capture the most relevant information for a particular task. The study utilizes PCA and a general regression neural network (GRNN) to create a gesture recognition system, successfully reducing redundant information and signal dimensions in surface EMG signals, which improves recognition efficiency and accuracy, ultimately achieving a 95.1% overall recognition rate and an average recognition time of 0.19 seconds [102]. In conclusion, unsupervised learning techniques offer the distinct advantage of discovering unexpected patterns and can be indispensable when labeled data is hard to come by. Their flexibility in dealing with unlabeled data opens doors to exploratory data analysis and the chance to identify novel data structures. However, they are not without challenges. Sensitivity to initial conditions, hyperparameter choices, and the necessity to understand the underlying data distribution are among the potential pitfalls [103]. Nevertheless, the demand for these techniques has been growing in situations where it is impractical to obtain large amounts of labeled data.

Overall, while each of these traditional machine-learning techniques has its advantages and limitations, their combined strengths have been leveraged in recent trends, leading to the emergence of hybrid models and ensemble methods. Such advancements aim to overcome the individual limitations of each technique and improve the robustness and accuracy of hand-motion recognition systems.

### Deep-learning techniques

Distinct from traditional supervised or unsupervised learning methods, convolutional neural networks (CNNs) are exceptionally equipped to autonomously learn spatial hierarchies of features, from simple edges to complex structures, making them particularly effective for tasks involving structured grid-data-like images. Their innate ability to automatically extract features from raw data without the necessity for manual feature engineering is a compelling advantage over conventional algorithms. Moreover, their depth allows them to represent intricate patterns that might be non-linearly separable, a feat tough for traditional methods [104]. They are ideal for image-based gesture-recognition tasks. The structure of a CNN includes an input layer, an output layer and various hidden layers that consist of convolutional layers, pooling layers and fully connected layers. Convolutional layers employ learnable filters to execute convolution operations, identifying local patterns within the data, such as spatial relationships between image pixels. Pooling layers, meanwhile, help reduce the spatial dimension of the representation, promoting network invariance to minor translations. Finally, the fully connected layers contribute to high-level reasoning based on the features previously extracted by other layers [105]. As exhibited in Fig. 5b, a novel study leveraged a CNN architecture based on LeNet-5, integrated with unique unimodal sensors, to accurately classify intricate finger and wrist motions. The system utilized an integrated unimodal sensor within a glove or sleeve, effectively distinguishing complex motions like tensioning, bending, shearing and twisting. This system achieved a mean classification accuracy of up to 90.2% for finger movements. Furthermore, this technology was utilized in a virtual text-entry interface system, converting specific finger movements into corresponding characters [106]. This accomplishment underscores the ability of CNN to maintain spatial context and automate hierarchical feature extraction, thereby eliminating the need for manual feature extraction. In a separate study, a model was trained successfully to recognize hand gestures from sensor data gathered by a smart glove, achieving an impressive recognition accuracy of 96.07% [107]. In another investigation, an optimized skeletonization algorithm in conjunction with CNNs was used for gesture recognition, thereby minimizing the impact of shooting angle and environmental factors, resulting in a recognition accuracy of 96.01% [108]. This result surpasses traditional methods like SVMs and dictionary learning with minimalistic representation. Furthermore, a study showcased the integration of a unique seamless multimode sensor with CNNs, accurately classifying (97.13% precision) various joint motion states. This demonstrates the effectiveness of CNNs in extracting and interpreting complex motion characteristics from sensor data [13].

Despite their strengths, CNNs do pose several challenges. They require significant amounts of annotated data for effective training, a process that can be both resource intensive and computationally demanding, particularly with deeper networks and larger data sets [109]. These limitations were apparent in a study that proposed a hand-motion recognition system based on thermal imaging with high resolution, which, while successful, demanded the creation of a substantial data set of 14 400 thermal hand postures and significant computational resources [110]. Furthermore, the computational requirements can be extensive, particularly with deeper networks and larger data sets. Finally, the decision-making processes and the features derived from CNNs can be complex and difficult to decipher, earning them the ‘black box’ moniker due to their lack of interpretability [111].

Recurrent neural networks (RNNs), including their variants such as long short-term memory (LSTM) networks, are a type of neural network architecture that is tailored to handle sequential data [112]. This makes them suitable for gesture-recognition tasks where temporal dependencies play a significant role. Unlike traditional feed-forward neural networks, RNNs possess cyclic connections, which allow them to maintain a ‘memory’ of previous inputs in the sequence [113]. This characteristic makes them uniquely capable of handling data where the order of inputs matters. Illustrating this capability, a recent study found that a multilayer RNN with an LSTM module outperformed traditional algorithms in gesture recognition, delivering superior speed and accuracy [114]. One popular variant of RNNs is LSTM networks. These networks incorporate a memory cell and gating units, enabling them to learn long sequences without suffering from issues like the vanishing or exploding gradient problem, common in standard RNNs. The key advantage of RNNs, particularly LSTMs, lies in their ability to learn from the temporal dependencies in data, which makes them adept at handling gestures with variable durations [115]. In the study, the LSTM network demonstrated its ability to decode complex hand movements through its application within a novel electronic skin system, as shown in Fig. 5c. This single skin sensor system, integrated with an LSTM network, recognized temporal patterns in the sensor signals in order to decode hand movements, illustrating the strength of LSTM in handling sequential data. Moreover, the researchers designed a rapid situation learning system to accommodate variations between users, underscoring the adaptability and effectiveness of the LSTM network in dynamic real-world scenarios [58]. In another study, the LSTM-based network demonstrated robust performance in classifying EMG patterns into six grip gestures at different force levels, leveraging its proficiency in time-series muscle contraction data crucial for nuanced hand-gesture recognition [116]. Despite the effectiveness of LSTM networks in processing time-series data, they can encounter difficulties with very long sequences due to their sequential nature. This can also result in slower training times compared to other architectures like CNNs. Theoretically, while LSTMs can capture long-term dependencies, in practice, they may struggle due to the complexity of the dependencies or learning challenges [117]. Furthermore, these networks require large volumes of labeled data and significant computational resources, and their decision-making processes are not easily interpretable [112]. Moreover, as evidenced in a study focusing on electroencephalography (EEG) signal classification for hand movements, LSTMs currently rely on hand-crafted features, which is a limitation that future work aims to address with more advanced feature extraction techniques [118].

Recent advancements in deep learning for gesture recognition include the combination of CNNs and RNNs, often known as convolutional recurrent neural networks (CRNNs). They exemplify the robust spatial feature extraction capabilities of CNNs and the sequence processing strength of RNNs. This synthesis makes CRNNs potent tools for tasks where both spatial and temporal data features are pivotal. Their integration provides a richer and more comprehensive understanding of gesture data than traditional algorithms that might separately process spatial and sequential data [119,120]. For example, one study demonstrates the effectiveness of a hybrid CNN-LSTM model in wrist kinematics estimation, utilizing a CNN for automatic feature extraction from sEMG data, and LSTM for sequence regression to account for temporal dependencies, thereby outperforming conventional CNN, LSTM and other machine-learning approaches in both intra-session and inter-session evaluations [121]. There is also an ongoing trend toward developing more efficient and lightweight deep-learning models to meet the demands of real-time gesture recognition on devices with limited computational resources, such as smartphones and wearable devices [122].

### Transfer and adaptive learning

Transfer and adaptive learning are machine-learning strategies that have seen increased application in the field of gesture recognition. They offer flexibility and efficiency in learning from high-dimensional data, rapidly generalizing from limited examples, and adapting to new situations.

Transfer learning involves using a pre-trained model, typically trained on a large-scale data set for a related task, and fine-tuning it for gesture recognition [123,124]. A typical example is the utilization of CNNs pre-trained on ImageNet, a substantial image classification data set [125]. These pre-trained models, having learned a diverse set of features from a vast number of images, can prove beneficial for differentiating various hand postures [126]. Utilizing transfer learning, this study efficiently applies pre-trained image classification networks like AlexNet and ResNet-18 to classify sEMG images, outperforming traditional machine-learning methods and achieving remarkable accuracies of 99.64% and 99.55% on the Rami data set, respectively, thereby demonstrating the potent impact of transfer learning in gesture-recognition tasks [127]. The advantages of transfer learning include reduced computational needs, faster training times and improved model performance, particularly when the available labeled data for the target task is limited [128]. However, the effectiveness of transfer learning may decrease if the source and target tasks are not closely related [129].

Adaptive learning refers to algorithms that can update their parameters over time, allowing them to adapt to new data or changing conditions. This technique is especially pertinent in gesture recognition due to the inherent variability in human gestures. Active learning is one key adaptive learning technique, where the learning algorithm strategically selects the most informative examples for labeling [130]. This approach can be valuable in scenarios where labeled data is scarce or expensive to obtain, such as in gesture recognition where obtaining labeled gesture data can require substantial manual effort [131]. Meta-learning, or ‘learning to learn’, is another essential example of adaptive learning. In meta-learning, the algorithms learn the strategy to learn from a variety of tasks, thereby allowing them to quickly adapt to new tasks with minimal examples [132]. An example of a meta-learning algorithm is model-agnostic meta-learning, which learns a model initialization that can be rapidly fine-tuned to a new task [133,134]. As depicted in Fig. 5d, researchers combined a substrate-less nanomesh receptor with an unsupervised meta-learning framework to successfully recognize different hand tasks, akin to human cutaneous receptors. They devised a time-dependent contrastive learning algorithm to differentiate unlabeled motion signals, thereby overcoming the limitations of traditional supervised learning models. This breakthrough showcases the potential of meta-learning in fields such as robotics, metaverse technologies and prosthetics, aligning with the principles of algorithms like meta-learning, renowned for their rapid fine-tuning adaptability [21]. The advantages of adaptive learning include the ability to cater to the personalized and dynamic nature of human gestures, thereby providing a more intuitive user experience. However, designing adaptive learning systems can present challenges such as the need for efficient update mechanisms, maintaining user privacy and ensuring data security [135,136].

The integration of transfer and adaptive learning signifies a recent trend that combines the ability to leverage pre-existing knowledge from large-scale pre-trained models and the capacity to adapt to new situations or individual variations. This trend is primarily driven by the rising prevalence of wearable technology and the continuous need for personalized and efficient gesture-recognition systems. Despite the challenges, the potential for improved and personalized gesture recognition offered by these techniques holds great promise for the future.

Supplementary Table S1 summarizes the commonly used machine-learning techniques and algorithms for recognizing hand motions using wearable soft sensors, delineating their advantages and limitations. This comprehensive overview illustrates a subtle correspondence between distinct methodologies and the scenarios they best serve. Supervised learning excels in tasks like basic gesture classification, reliably discerning straightforward hand movements when backed by a rich set of labeled data sets. However, as the complexity of gestures increases, the accuracy of the model can be challenged, emphasizing the necessity of diverse and representative training samples. In contrast, unsupervised learning provides an innovative approach to identifying emerging gestures, especially when exploring untapped user segments. Yet, in the absence of labeled benchmarks, its results can sometimes be ambiguous, underscoring the importance of meticulous validation techniques. CNNs excel in situations demanding intricate signal processing, particularly when interpreting multidimensional data from wearable sensors for hand-pose recognition. While their capacity to process visual information is unparalleled, they often necessitate an extensive volume of labeled data. Moreover, the intricate architecture of CNNs can sometimes present challenges in interpretation and fine-tuning. RNNs and LSTMs, designed to excel with time-series data, emerge as frontrunners for endeavors such as continuous sign language interpretation. Their sequential nature, however, can sometimes struggle when faced with extensive motion sequences, necessitating careful model tuning. The fusion of CNNs and RNNs gives rise to hybrid models, which offer enhanced capabilities in endeavors like real-time sign language translation. While this synergy captures both spatial and temporal nuances effectively, it also introduces added complexity, potentially increasing the computational burden. Transfer learning is valued for its adaptability, allowing models to migrate efficiently from one device environment to another. This flexibility makes it ideal for environments where devices or contexts frequently change. However, caution is needed due to potential biases originating from the primary training environment. Adaptive learning, known for its continual optimization, excels in personalized gesture recognition, adapting to the unique nuances of individual users over time. However, despite its promise, its dynamic character could mean regular refinements are necessary to maintain accuracy.

It is imperative to emphasize that while these scenarios have been highlighted as illustrative applications for each method, they are not prescriptive. The ideal algorithm for a specific task might vary due to numerous influencing elements, including data integrity, technical limitations and user inclinations, among others [137]. To conclude, hand-motion recognition spans a wide range of algorithms, each marked by its strengths and challenges [138]. In understanding this domain, recognizing the intricate alignment between algorithms and their suitable applications is vital.

## BRIEF RESEARCH HISTORY OF HAND-MOTION TRACKING USING WEARABLE SOFT SENSORS

**Figure 6.**
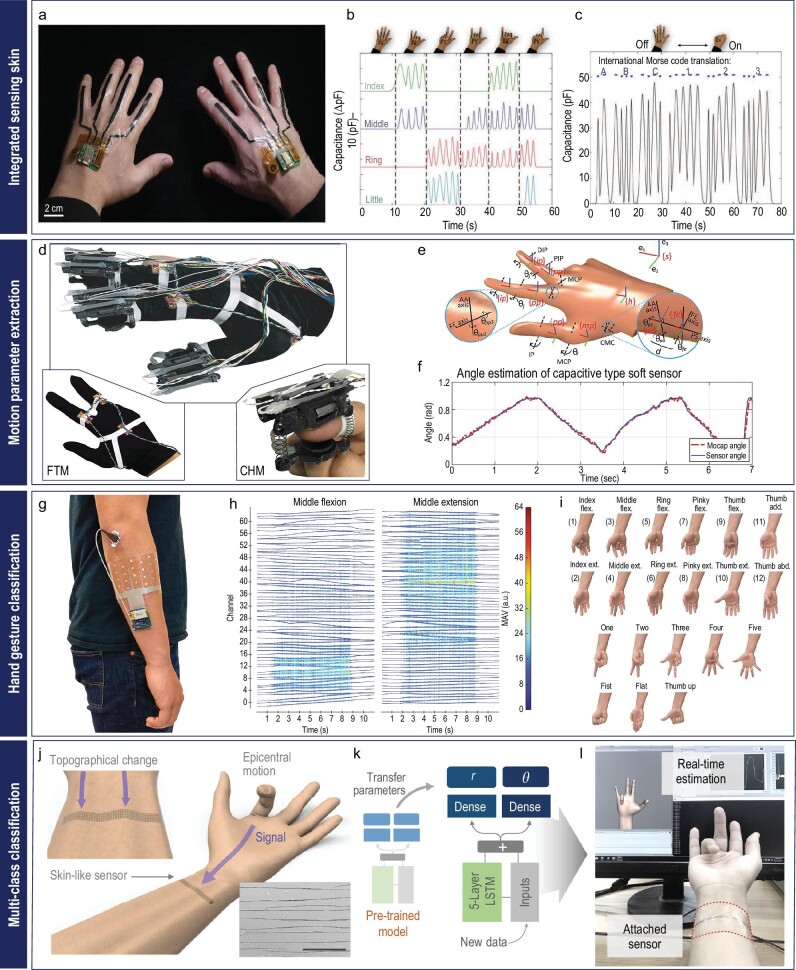
Brief development history of the wearable soft sensor for hand-motion tracking. (a-c) Wearable sensing skin for hand-motion tracking without AI technology: (a) optical image of sensing skin with commercially available conducting acrylic tape mounted on a human hand (scale bar: 2 cm). Reproduced with permission from ref. [139]; copyright 2016 John Wiley and Sons. (b) The sensor signals on each joint, corresponding to different hand motions. Reproduced with permission from ref. [139]; copyright 2016 John Wiley and Sons. (c) Morse code created by manipulating hand gestures (opening and closing) with different time intervals. Reproduced with permission from ref. [139]; copyright 2016 John Wiley and Sons. (d-f) Finger angle estimation: (d) digital image of the gloves equipped with soft sensors for finger tracking. Reproduced with permission from ref. [142]; copyright 2019 IEEE. (e) Bending angle analysis for joints, and links in the coordinate system. Reproduced with permission from ref. [142]; copyright 2019 IEEE. (f) The estimation result of finger bending angle and comparison with reference sensor. Reproduced with permission from ref. [142]; copyright 2019 IEEE. (g-i) Wireless sensor array for hand-gesture recognition: (g) photograph of the screen-printed sEMG sensor array on a human arm. Reproduced with permission from ref. [146]; copyright 2021 Springer Nature. (h) The sEMG data of 64 channels gathered during human-gesture recognition. Reproduced with permission from ref. [146]; copyright 2021 Springer Nature. (i) Various hand gestures recognizable from the sEMG sensor array. Reproduced with permission from ref. [146]; copyright 2021 Springer Nature. (j-l) Estimation of the five finger motions in a single sensor: (j) graphical illustrations of skin-like sensors that decode signals induced by topographical change (inset: SEM image of the crack induced by UV laser, scale bar: 40 μm). Reproduced with permission from ref. [58]; copyright 2020 Springer Nature. (k) Strain mapping of the forearm during different bending motions of fingers (top) and corresponding metric space (bottom). Reproduced with permission from ref. [58]; copyright 2020 Springer Nature. (l) Real-time estimation of the five different finger motions on the screen. Reproduced with permission from ref. [58]; copyright 2020 Springer Nature.

During the initial stages of hand-motion tracking, various attempts were made to capture signals from sensors located on gloves or human hands. For example, wearable sensing skin for hand-motion tracking was fabricated using commercially available conductive acrylic tape as the sensing material (Fig. 6a) [139]. Employing laser patterning, the conductive tape was shaped into the desired configuration and seamlessly integrated into a wireless unit. To achieve hand-motion detection, conductive tape-based sensors were strategically attached to each finger. The wearable sensing skin was a pioneer in the generation of signals based on hand movements (Fig. 6b). However, since the generated signals roughly represented hand movements and only contained information about whether they were bent or not, it was limited to performing simple tasks like Morse code communication through the manipulation of hand gestures with varying time intervals (Fig. 6c). Similarly, gloves with carbon nanotube (CNT) strain sensors and Ag NW-coated polyvinylidene fluoride-trifluorethylene (PVDF-TrFE) fiber-based strain sensors on every finger joint, stably generated electromechanical signals regarding finger movements [140,141].

Building upon this, to achieve more precise tracking of hand movements, several studies have been conducted to predict bending angles in a 3D space. By opportunistically utilizing various types of sensors, including inertial measurement units, force-sensitive resistor sensors and soft sensors (Fig. 6d), the finger-motion tracking device demonstrated that complex, anatomically consistent multi-degree-of-freedom (DOF) finger motions could be tracked, as demonstrated in Fig. 6e and f [142]. Furthermore, a finger-motion tracking system in a 3D space was developed using a microfluidic strain sensor [143]. This study introduces algorithms for detecting diverse non-coplanar movements, such as abduction/adduction and flexion/extension joint angles, through the tracking of the first metacarpal bone in various thumb positions. By strategically positioning sensors, it becomes possible to achieve joint angle measurements with an accuracy of <3.5 degrees. In addition, a resistive strain sensor utilizing liquid metal was fabricated through direct ink-printing [144]. The popular liquid metal, eutectic gallium-indium (EGaIn), was patterned into glove-type sensors for strain measurement, allowing the bending angle of each joint to be assessed using a mock-up hand. The high-resolution direct ink-printing technique facilitated the creation of two sensors on each finger. By utilizing the electrical signals from these two sensors, joint angles were measured and finger postures were visualized in real time through animated representation.

However, the integration of deep neural networks enables the detection of more complex hand motions with the utilization of capacitive sensors consisting of a silicone-based dielectric layer sandwiched between two conductive layers made from silicone mixed with carbon black [145]. These sensors were laser cut into a hand shape, resulting in an array of 44 individual sensors that were subsequently attached to a glove. By employing a CNN with the sensor array, bending angles were predicted with 92% accuracy and mean errors below 15 degrees. Moreover, the glove equipped with the CNN demonstrated rapid and accurate real-time prediction of diverse hand poses.

A different hand-motion tracking system was developed utilizing sensor arrays to track hand motion through sEMG measurements (Fig. 6g) [146]. Commercially available conductive Ag ink was screen printed onto a polyethylene terephthalate (PET) substrate, and subsequent photonic sintering processes enhanced ink adhesion. This allowed the capture of sEMG signals from the flexor and extensor muscles associated with finger movement when attached to the forearm. The sEMG data from 64 individual sensors were wirelessly transmitted and converted into different hypervectors corresponding to various hand gestures (Fig. 6h). Utilizing a hyperdimensional computing algorithm, the system successfully classified 21 hand gestures with 92.87% accuracy, regardless of the number of fingers involved in the motion (Fig. 6i).

While the sensor array system demonstrated highly accurate prediction performance, a crucial aspect of wearable soft sensors is reducing the number of sensors while maintaining performance. This reduction enables a smaller and less computationally intensive system. Addressing this need, a single-sensor skin sensor was designed to decode and interpret human motions (Fig. 6j) [58]. The authors induced cracks on sintered Ag nanoparticles (NPs) using UV lasers, allowing for the detection of subtle skin deformations and muscle movements with high sensitivity. Subsequent laser ablation techniques were employed to create serpentine-shaped sensors, enabling better conformality to the skin. Using the LSTM network, the authors successfully created a metric corresponding to the bending of each finger and matched it with signals from the sensor. Furthermore, rapid situation learning (RSL) algorithms utilizing transfer learning techniques facilitated adaptation to different positions on the wrist and significantly reduced retraining time for new users (Fig. 6k). As a result, a serpentine-shaped single-crack sensor coupled with an LSTM network achieved finger motion classification with 92.6% accuracy when attached to the human wrist (Fig. 6l).

As summarized in this section, soft sensors have made significant contributions and advancements in tracking hand gestures over the past decade. As a result, current technology has reached a level where it can classify and predict specific types of hand motions in real time, opening up numerous possibilities for various applications. However, to achieve a level of technology that can accurately predict hand gestures in real time, there is a need for the capability to extract quantitative and dynamic parameters such as angular velocity and angular acceleration. Furthermore, to move beyond laboratory-level research and advance into real-world technology, several key challenges need to be addressed. These challenges include the ability to capture the necessary ‘true signals’ for applications from the complex and massive signals generated by various hand gestures in a natural environment. From the next section onwards, we will introduce promising applications enabled by hand-gesture recognition.

## HAND-GESTURE-RECOGNITION-BASED APPLICATIONS IN OUR DAILY LIVES

As previously mentioned, the integration of soft sensors and AI technology holds great potential for real-life applications, particularly in extracting specific signals from large volumes of real-time random data. This capability is essential for rapid and accurate estimation of hand motion, thereby enhancing human–machine interfaces. In the following section, we will explore diverse practical applications that are made possible through the integration of wearable soft electronics and machine-learning algorithms.

### Machine control

Wearable soft sensors have emerged as a viable substitute for conventional remote controllers, granting humans mobility and freedom of movement while interacting with machines. Unlike remote controllers, these sensors capture the movements of users and gestures, facilitating a more intuitive and natural interaction. For instance, controlling the flight path of a drone can be accomplished simply by moving human arms, offering a more intuitive alternative to using buttons or joysticks on a remote controller [85,147].

**Figure 7.**
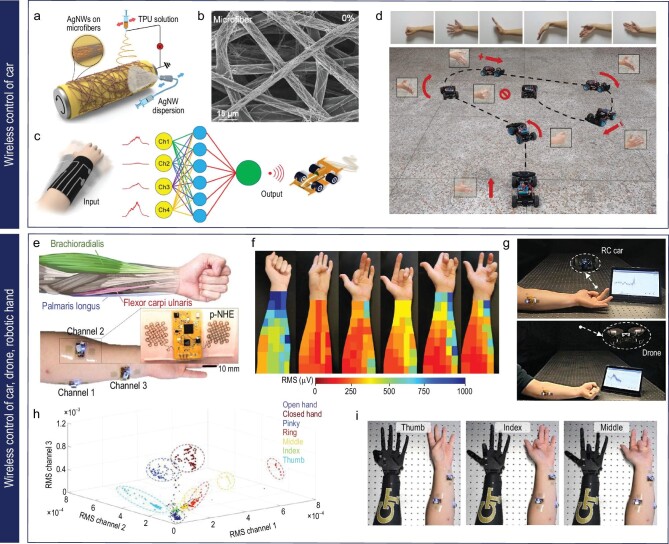
Hand-gesture-based machine control system. (a-d) Wireless hand-gesture-based control of a car enabled by a car arm sleeve: (a) schematic image of the fabrication process for electrospun TPU fibers and spray-coated AgNW-based conductive textiles. Reproduced with permission from ref. [17]; copyright 2021 American Chemical Society. (b) SEM image of conductive textiles consisting of AgNWs and TPU microfibers (scale bar: 15 μm). Reproduced with permission from ref. [17]; copyright 2021 American Chemical Society. (c) Graphical images of a wirelessly controlled car driven by 4-channel signals with machine learning. Reproduced with permission from ref. [17]; copyright 2021 American Chemical Society. (d) Optical images of the path of a car guided wirelessly by six hand gestures. Reproduced with permission from ref. [17]; copyright 2021 American Chemical Society. (e-i) Wireless hand-gesture-based control of a car, drone and robotic hand: (e) graphical illustration of the three representative forearm muscles (top) and a digital image of the location of the graphene-based device to measure EMG data of the muscles (bottom). Reproduced with permission from ref. [18]; copyright 2020 Springer Nature. (f) Digital image of the EMG mapping data depending on six-finger motion. Reproduced with permission from ref. [18]; copyright 2020 Springer Nature. (g) Optical images of wireless hand-gesture control by the single device on the forearm. Reproduced with permission from ref. [18]; copyright 2020 Springer Nature. (h) Classification of seven hand gestures using EMG data from three devices and the CNN model. Reproduced with permission from ref. [18]; copyright 2020 Springer Nature. (i) Photographs of a robotic hand that shadows human hand motion. Reproduced with permission from ref. [18]; copyright 2020 Springer Nature.

In this context, a smart arm sleeve that consists of spray-coated Ag NWs on a thermoplastic polyurethane (TPU) electrospun fiber matrix serves as a human–machine interface, allowing control commands to be acquired through hand gestures (Fig. 7a) [17]. The porous microstructures of conductive textiles offer soft mechanical properties, high vapor permeability and excellent washability for long-term wearable use (Fig. 7b). The sleeve incorporates a four-channel sensing system sewn onto an elastomeric microtextile, providing comfortable and stable contact with the skin (Fig. 7c). By adjusting the electrodes to key muscles in the forearm, the sleeve captures high-fidelity signals reflecting different hand gestures. To identify and categorize these gestures, a machine-learning algorithm called extreme gradient boosting (XGboost) is employed, allowing the correlation of voltage signals with specific control commands. XGboost mimics the cognitive learning ability of the human brain by analyzing characteristic features extracted from training data sets composed of EMG signals associated with standard hand gestures. By establishing explicit correlations between complex voltage signals and corresponding control commands, the XGboost algorithm enables accurate identification and categorization of distinctive gestures. This gesture-recognition system enables wireless control of a four-wheeled car using standard hand gestures, demonstrating the potential of smart human–machine interfaces (Fig. 7d).

Another target machine that utilizes hand gestures is a robotic hand designed to mimic and replicate the hand movements of the user [148]. The integration of robotic hand technology in a wireless manner offers numerous beneficial aspects in our daily lives. By combining telemedicine, wearable soft sensors with AI technology and robotic hands, remote patient monitoring, diagnosis and treatment become more accessible, reducing the need for in-person visits, especially for routine check-ups and chronic condition management. For this objective, a wireless, flexible and interconnected electronic system using multiple layers of graphene was designed [18]. The system incorporated a functionalized conductive graphene material, known for its improved biocompatibility, oxidation resistance and ability to be soldered, which played a crucial role in enabling the flexibility of wireless circuit. In order to detect various classes of control, three of the created devices were affixed to three muscles with physiological significance: palmaris longus, brachioradialis and flexor carpi ulnaris (Fig. 7e). The use of high-aspect-ratio graphene enabled gel-free and precise recording of muscle activities through EMG data mapping (Fig. 7f). Wireless real-time machine control was demonstrated using EMG signals collected by the printed electronics. The authors employed CNN machine-learning techniques for the accurate classification of acquired EMG data. The CNN algorithm consisted of a two-layer network, and the authors achieved a high classification accuracy of over 99% for six classes in real-time control. At first, with a single device on palmaris longus, the authors successfully wirelessly controlled the drone and RC car (Fig. 7g). Furthermore, the researchers created a 3D, three-channel root mean square (RMS) plot that showed seven distinctive clusters corresponding to individual finger motions and hand gestures (Fig. 7h). By distributing the three devices over different muscle groups, they successfully classified seven classes with 98.6% accuracy. The authors demonstrated the application of their wearable devices by controlling a robotic hand through wireless, real-time control (Fig. 7i). The study highlights the potential of these devices for various portable human–machine interface applications, such as controlling humanoid robots, drones, prosthetic hands, display interfaces and electronic wheelchairs. In another study, a self-powered TENG-based smart bandage to control a robotic hand was proposed [149]. This innovative bandage, composed of multi-walled CNTs, silica gel and a soft adhesive bandage, effectively captured various human movements such as finger movements, elbow gestures and leg motions, as well as breathing and swallowing patterns. By attaching this bandage to the forearms and utilizing LSTM algorithms, the authors successfully distinguished three hand gestures and enabled the robotic hand to mimic the movements of the user accordingly.

### Sign-to-language translation and text-input systems

**Figure 8.**
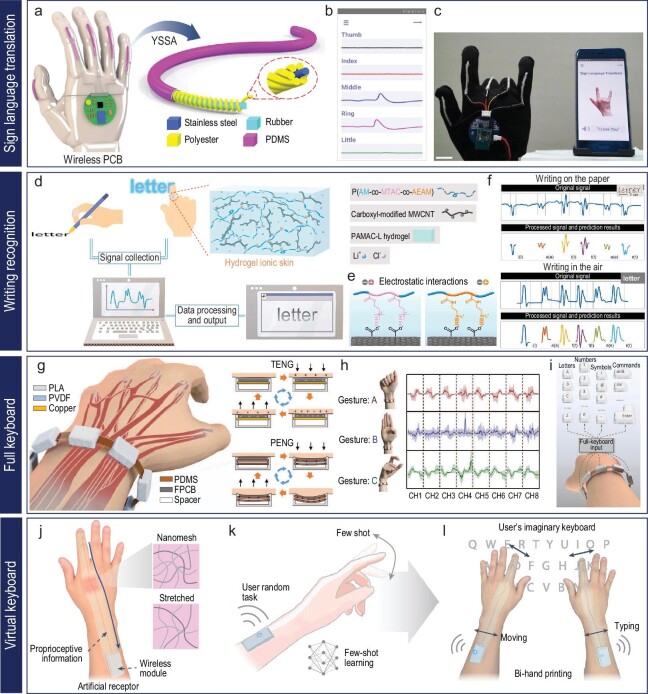
Text input systems. (a-d) Sign language translation: (a) schematic images of a YSSA and YSSAs attached to five fingers and PCB for wireless data acquisition (left), and schematic illustrations of the materials and structure of the YSSA (right). Reproduced with permission from ref. [19]; copyright 2020 Springer Nature. (b) Five sensor signals corresponding to the movement of each finger. Reproduced with permission from ref. [19]; copyright 2020 Springer Nature. (c) Photograph of the sign language translation using a YSSA. Reproduced with permission from ref. [19]; copyright 2020 Springer Nature. (d-f) Writing recognition: (d) schematic images of writing recognition with hydrogel ionic skin. Reproduced with permission from ref. [150]; copyright 2022 Elsevier. (e) Schematic illustration of the ionic interactions in the skin. Reproduced with permission from ref. [150]; copyright 2022 Elsevier. (f) Signal of the ionic skin and prediction result of during hand writing on the paper (top) and in the air (bottom). Reproduced with permission from ref. [150]; copyright 2022 Elsevier. (g-i) Self-powered hand-gesture tracking wristband for sign language translation and keyboard input system: (g) schematics of a self-powered wristband and its working mechanism (hybrid system of TENG and PENG). Reproduced with permission from ref. [20]; copyright 2022 John Wiley and Sons. (h) Representative three hand signs and their corresponding eight sensor signals from sensors on the wristband. Reproduced with permission from ref. [20]; copyright 2022 John Wiley and Sons. (i) Schematic image of the keyboard input system with the wristband. Reproduced with permission from ref. [20]; copyright 2022 John Wiley and Sons. (j-l) Virtual keyboard realized by substrate-less nanomesh for the recognition of finger motion: (j) schematics of biocompatible substrate-less nanomesh and wireless module (inset: schematics of the nanomesh during finger motion). Reproduced with permission from ref. [21]; copyright 2023 Springer Nature. (k) Graphical photography of the signal generation and wireless transmission for meta-learning for few-shot learning. Reproduced with permission from ref. [21]; copyright 2023 Springer Nature. (l) Illustration of the virtual keyboard enabled by recognition of finger movements with nanomesh on two hands. Reproduced with permission from ref. [21]; copyright 2023 Springer Nature.

Soft electronics with hand-gesture recognition functions can play a vital role in sign-to-language translation systems by bridging the gap between sign-language users and those who rely on written language. They enable individuals proficient in sign language to convert their signed messages into written text, facilitating effective communication with a broader audience. For example, an innovative sign-to-speech translation system was created, employing a yarn-based stretchable sensor array (YSSA), which can be worn as a wearable device (Fig. 8a) [19]. The YSSA consisted of a core rubber microfiber coiled around conductive yarns, comprising an outer polyester layer and an inner stainless-steel layer. These conductive yarns were encased in a PDMS sleeve, enabling the generation of electrical signals through triboelectrification and electrostatic induction. The YSSA comprised five channels, each generating distinct electrical signals corresponding to the movement of the five fingers (Fig. 8b). These signals were wirelessly recorded via a Bluetooth module. By applying SVM algorithms, the YSSA successfully classified 11 hand gestures with an accuracy of 98.63%. Furthermore, the hand gestures representing sign languages were effectively translated into corresponding letters (Fig. 8c).

Beyond sign-to-language translation, the integration of soft electronics in writing-recognition systems and virtual keyboards, capable of capturing and interpreting human hand motion, holds the potential to significantly enhance accessibility for individuals with physical disabilities. In addition, text input is a valuable substitute for sign language, serving as a universal medium for communication and facilitating written documentation. By substituting conventional physical keyboards, these writing recognition systems and virtual keyboards can offer an alternative input method that facilitates effective communication, particularly for individuals facing challenges with traditional physical keyboards.

In this respect, a handwriting recognition system utilizing a hydrogel ionic skin was developed (Fig. 8d) [150]. The hydrogel, denoted as PAMAC-L hydrogel, was synthesized using a copolymer composed of acrylamide (AM), trimethylammonium chloride (MTAC) and 2-aminoethyl acrylamide hydrochloride (AEAM), along with carboxyl-modified multi-walled CNT fillers and LiCl. Incorporating a limited quantity of multi-walled CNTs to serve as a mechanical reinforcement agent ensured the mechanical robustness of the hydrogel through binary interfacial electrostatic interactions with the copolymer network, all while maintaining transparency (Fig. 8e). Furthermore, the addition of a small amount of LiCl salt ions to the hydrogel significantly increased its electrical conductivity without compromising mechanical robustness and biocompatibility. When attached to finger joints, the piezoresistive hydrogel ionic skin consistently generated relative resistance change signals corresponding to handwriting gestures, whether performed on paper or in the air (Fig. 8f). By employing the bagged tree ensemble machine-learning algorithm, the handwriting-recognition system achieved successful recognition of individual letters as well as short sentences, both in the physical writing and air-writing scenarios.

Additionally, a machine-learning-based smart gesture-recognition wristband that can act as a full keyboard and multi-command input is developed (Fig. 8g) [20]. The smart wristband recognizes sign languages, translates it in real time and broadcasts the translated sign language as voice. The wristband consists of eight hybrid generators with TENG and PENG, which are respectively used to differentiate the large force and slight contact, enabling the precise classification of hand gestures. A single sensor is composed of a PDMS layer, PVDF layer and copper layer for TENG, PENG and electrode, respectively, which are directly connected to a flexible printed circuit board (PCB). Moreover, the system is designed to wirelessly transmit the mechanical information gathered by the hybrid sensor via Bluetooth module. The eight hybrid sensors produced distinctive electrical signals depending on the movement of the hand (Fig. 8h). However, the hybrid sensors on the wristband could not produce exactly the same signals due to the different wearing states and types of users. The machine-learning technique effectively solves the problems caused by different users and the randomness of the wearing state, by relearning the gestures of a new wearer. The linear discriminant analysis (LDA) model successfully recognizes 26-letter gestures with 92.6% accuracy. The wristband can translate the sign language into corresponding letters, meaning that it acts as a full keyboard, with hand gestures used to create sentences (Fig. 8i).

Furthermore, a novel virtual keyboard technology was developed by utilizing biocompatible nanomesh integrated with Ag-Au core-shell NWs (Fig. 8j) [21]. This innovative approach allows direct spray coating of the biocompatible NWs on human skin, eliminating the need for conventional polymeric substrates that often lead to issues such as delamination and lack of conformality and breathability. Consequently, this substrate-less property enables the sensor to capture intricate signals with exceptional sensitivity, generating distinguishable electrical signals from various gestures using a single sensor. The captured information regarding electrical resistance changes is wirelessly transmitted to a computer through a silicone elastomer module with a built-in battery. To enhance adaptability to different users and tasks, a system leveraging few-shot meta-learning was implemented (Fig. 8k). This system generates a separable latent feature space specifically designed for hand-gesture recognition, enabling improved performance across diverse user profiles and tasks. By utilizing unlabeled random motion data, a learning model was developed to project signals into this separable space, resulting in rapid and accurate gesture classification, even for users not present in the training data set. The authors collected finger motions using the substrate-less nanomesh and employed a time-dependent contrastive (TD-C) network to extract valuable motion features, effectively representing joint signals and enabling the translation of skin stretches into multi-joint proprioception. To demonstrate the capabilities of their learning framework, the authors conducted experiments involving one-handed numpad typing and two-handed sentence typing (Fig. 8l). Remarkably, they achieved an 85% accuracy rate while typing several sentences using an imaginary keyboard. These results showcase the ability of the system to effectively decode a wide range of finger motions and recognize gestures, making it highly suitable for virtual keyboard applications.

### Object recognition and its extension to AR/VR applications

Object recognition is a fundamental technology that is necessary for wearable soft systems as it enables seamless and intuitive interaction between humans and machines. Conventional soft sensors have proven their efficacy in measuring elementary mechanical information such as strain, bending angle and pressure. However, with the integration of AI technology, soft sensors equipped with object recognition capabilities empower wearable soft systems to comprehend and respond to their surrounding environments in real time.

**Figure 9.**
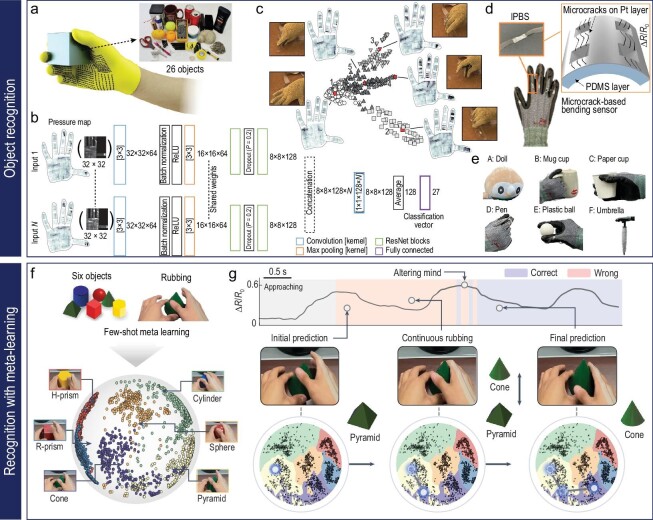
Object recognition. (a-e) Gloves for object recognition: (a) photographs of gloves with 548 sensors and 26 recognizable objects. Reproduced with permission from ref. [151]; copyright 2019 Springer Nature. (b) Structures and signal processing of the CNN model for object recognition. Reproduced with permission from ref. [151]; copyright 2019 Springer Nature. (c) Tactile maps of gloves during single-object interaction with different grasping postures. Reproduced with permission from ref. [151]; copyright 2019 Springer Nature. (d) Optical images of the bending sensor (IPBS) on the gloves and its working mechanism. Reproduced with permission from ref. [152]; copyright 2022 American Chemical Society. (e) Photographs of six objects used for the classification task. Reproduced with permission from ref. [152]; copyright 2022 American Chemical Society. (f-g) Object recognition with meta-learning: (f) object recognition by rubbing, with few-shot meta-learning (top) and a sphere diagram of six object recognitions (bottom). Reproduced with permission from ref. [21]; copyright 2023 Springer Nature. (g) Sensor signal (top) and the corresponding sequential changes of position in the projected embedding space (bottom) during the meta-learning process, by rubbing new objects. Reproduced with permission from ref. [21]; copyright 2023 Springer Nature.

In line with this objective, a tactile glove comprising an array of 548 sensors was developed to capture data pertaining to human grasping, providing comprehensive coverage of the entire hand (Fig. 9a) [151]. This tactile glove consisted of a custom-knit glove integrated with a sensing laminate. The sensing laminate was fabricated through laser-cutting a force-sensitive film to conform to the shape of the hand. Conductive thread electrodes were meticulously sewn on either side of the force-sensitive film, and the laminate was secured in place using stretchable adhesive tape, insulated with a thin low-density polyethylene film. To ensure insulation, the exposed conductive threads were coated with a PDMS layer, and connected to insulation-displacement connectors. To analyze and classify different types of grasps based on the tactile data obtained from the glove, the researchers employed machine-learning algorithms, specifically CNN learning (Fig. 9b). The CNN was trained on a substantial data set of grasp signatures, enabling it to learn the distinctive patterns and characteristics associated with various grasp types (Fig. 9c). The study successfully demonstrated the efficacy of the tactile glove and the machine-learning approach in identifying and classifying diverse grasp types, encompassing precision grips, power grips and lateral grasps. Consequently, the tactile glove exhibited the ability to successfully identify 26 frequently used objects in daily life.

In another study, a microcrack-based bending sensor utilizing a platinum (Pt) layer deposited on a PDMS substrate was developed for joint applications (Fig. 9d) [152]. The incorporation of inverse pyramid patterns on the PDMS substrate facilitated the controlled formation of microcracks, resulting in a highly linear response. The inverse pyramid-structured bending sensor exhibited remarkable potential for motion monitoring and tactile object recognition on human fingers. By deploying the sensors on individual fingers, the bending angles were tracked during the manipulation of six common objects (Fig. 9e). Notably, the implementation of machine-learning algorithms, namely random forest (with an accuracy of 92.2%) and gradient boosting classifiers (with an accuracy of 87.2%), facilitated the successful classification of the aforementioned objects.

In addition, a previously introduced biocompatible nanomesh integrated with a meta-learning system for a virtual keyboard demonstrated object recognition based on gestural information through surface rubbing, achieving an accuracy of 82.1% (Fig. 9f) [21]. The labeled data set was visualized on a sphere diagram, employing distinct colors to represent individual objects. Furthermore, the nanomesh with meta-learning algorithm demonstrated its learning ability (Fig. 9g). Initially, distinguishing between pyramid and cone shapes proved challenging, resulting in overlapping embedded points within the diagram. Consequently, the model initially misclassified pyramid interactions as cones. However, continuous rubbing facilitated a progressive learning process, enabling the model to gradually differentiate between the two shapes due to the adaption of few-shot meta-learning. Notably, the embedded vectors representing the objects transitioned from one region to another within the diagram, emulating the human-like process of incremental object recognition and perceptual adjustments during interactive experiences.

Recently, AR and VR technologies have garnered attention from the engineering society due to their potential to enhance visualization, improve training and simulation, and facilitate remote collaboration [153]. When it comes to AR and VR, object recognition technology enhances the experience of the user by seamlessly integrating virtual content with the real world. By recognizing and understanding the surrounding objects, AR/VR systems can overlay virtual objects or information onto the view of the user, creating an interactive and immersive environment. Soft electronics serve as suitable platforms for these technologies by virtue of their flexibility, conformability and seamless integration into wearable devices, thereby addressing limitations associated with traditional controllers for controlling virtual characters [154]. Traditional controllers have constraints in terms of expressible actions and simultaneous control of multiple movements. However, leveraging soft electronics enables users to experience greater freedom and flexibility in controlling virtual characters, fostering a more immersive and seamless interaction. Furthermore, the integration of AI technology into AR/VR applications holds vast potential for enhancing performance. Machine-learning algorithms can optimize character control, enhance realism and provide intelligent responses to user inputs, thus enriching the overall experience and blurring the boundaries between the real and virtual worlds. This integration creates new horizons for AR/VR applications, revolutionizing domains such as entertainment, training simulations and various other fields reliant on immersive virtual experiences.

**Figure 10.**
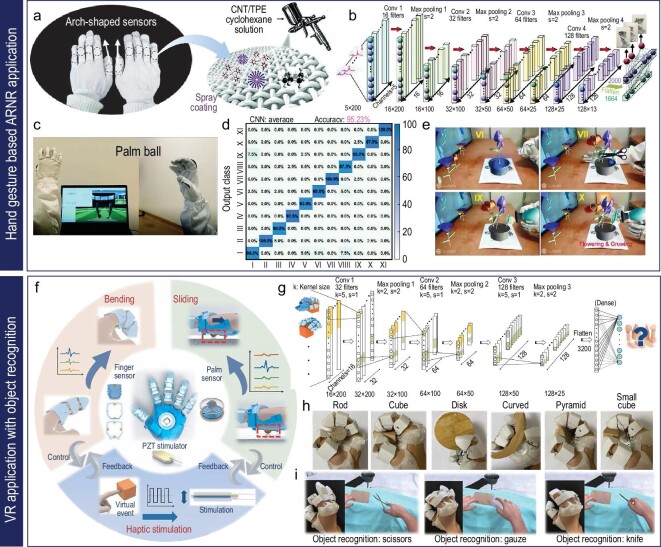
AR/VR applications. (a-e) Smart gloves for AR/VR applications: (a) photograph of smart gloves and schematics of spray-coated CNT/thermoplastic elastomer (TPE) composites on the smart gloves. Reproduced with permission from ref. [22]; copyright 2020 John Wiley. (b) Graphical illustrations of CNN model structures and their processes. Reproduced with permission from ref. [22]; copyright 2020 John Wiley. (c) Application of a smart glove for VR control in a VR baseball game environment. Reproduced with permission from ref. [22]; copyright 2020 John Wiley. (d) The confusion matrix of 11 class-hand gesture recognitions for an AR environment. Reproduced with permission from ref. [22]; copyright 2020 John Wiley. (e) Screenshots of AR control in AR space. Reproduced with permission from ref. [22]; copyright 2020 John Wiley. (f-i) Smart gloves for object recognition and AR/VR applications: (f) schematics of smart glove configuration and the working mechanisms of the finger sensor, palm sensor and PZT simulator. Reproduced with permission from ref. [23]; copyright 2020 American Association for the Advancement of Science. (g) Graphical photography of the process for establishing the CNN model. Reproduced with permission from ref. [23]; copyright 2020 American Association for the Advancement of Science. (h) Digital images of target objects and corresponding hand gestures. Reproduced with permission from ref. [23]; copyright 2020 American Association for the Advancement of Science. (i) Screenshots of smart gloves for a VR surgical training program based on object recognition. Reproduced with permission from ref. [23]; copyright 2020 American Association for the Advancement of Science.

A superhydrophobic triboelectric textile was employed to realize glove-based human–machine interaction in AR/VR environments (Fig. 10a) [22]. The authors applied a coating of CNTs and thermoplastic elastomer to achieve super-hydrophobicity and enhance triboelectric performance. This coating effectively addresses performance degradation caused by sweat and humidity, which can negatively impact triboelectric performance. CNNs are employed to recognize different gesture patterns based on triboelectric signals from the sensors (Fig. 10b). High recognition accuracy is achieved for different gestures, including those with similar signal patterns. Using the glove with a hand-gesture-recognition system, the authors can control the hand to determine the pitch in the baseball pitching game (Fig. 10c). Furthermore, the glove-based human–machine interface can also be applied to more complex gestures involving all 10 fingers with 95% accuracy (Fig. 10d), allowing various hand tasks related to flower arrangement in the AR application (Fig. 10e).

In addition, smart gloves incorporating biocompatible elastomer-based TENG sensors and lead zirconate titanate (PZT) piezoelectric mechanical haptic stimulators have been developed for applications in VR surgical training programs (Fig. 10f) [23]. The TENG sensors, resembling hemispheres, were fabricated through a straightforward process involving the pouring of elastomers into 3D-printed molds. These sensors were subsequently attached to the gloves to capture hand movements and external object contact information. Specifically, Ecoflex-based TENG sensors embedded on the fingers were designed to detect multi-DOF bending motions, while PDMS-based sensors on the palm were employed to sense normal and shear forces upon contact with external objects. Additionally, the PZT mechanical stimulator provided haptic feedback to simulate interactive events in the virtual reality environment. In this study, human skin served as the positive triboelectric material, generating triboelectric signals upon contact. The TENG sensors with CNN algorithms successfully translated these signals into distinctive voltage patterns, enabling the classification of various multidimensional hand gestures, including movement states, bending angles and object contact states (Fig. 10g). A key aspect of the VR surgical training program involved real-time object recognition facilitated by the integration of 16 TENG sensors on the fingers and palms, coupled with SVM algorithms for machine learning. The SVM algorithms achieved an accuracy of 91%. It should be noted that a three-layer CNN algorithm demonstrated superior performance (∼96% accuracy) to SVM algorithms, but the authors opted for SVM algorithms for the primary demonstration. Ultimately, the smart gloves successfully realized a VR surgical training program by incorporating diverse finger motions and hand positioning (Fig. 10h). Users were able to manipulate surgical instruments and switch control modes within the virtual reality environment (Fig. 10i). The integration of advanced sensing capabilities and haptic feedback mechanisms offers promising avenues for enhanced training experiences and realistic interactions in VR and AR applications.

## CHALLENGES AND OUTLOOK

The utilization of soft sensors for hand-motion tracking has witnessed remarkable growth. Current technologies have reached a point where they effectively combine soft sensors with machine learning to classify hand-motion types with high accuracy, showcasing potential applications. However, these advancements primarily remain at the laboratory level [155]. This limitation stems from the fact that current technologies deliberately distinguish signals required for application implementation in highly constrained hand-movement scenarios. While recognizing predefined hand movements in controlled settings seems feasible, the real challenge lies in adapting these methodologies to the dynamic and intricate landscapes of real-world environments [156]. In such natural environments, the generation of complex, random and extensive signal data significantly complicates the task of accurately recognizing hand motions. In this section, we will discuss several challenges and possible solutions that machine-learned soft sensors face on their path to practical real-world applications with hand-gesture recognition. These challenges encompass the realms of soft sensors, data collection and analysis for hand-motion recognition and machine learning.

### Mechanical robustness at the interface of different materials

Soft sensors employed for hand-motion recognition, typically integrated into gloves or worn on the human hand, are frequently subjected to mechanical deformation due to their intended usage. Among the challenges encountered in such scenarios, one of the most prominent is the mechanical failure of the device arising from repeated strains, resulting in unstable interconnections [157]. This issue predominantly stems from stress concentration at the interfaces between dissimilar materials [158]. Soft materials, characterized by their unique mechanical properties, distribute mechanical stress differently based on their individual traits. For instance, when rigid components such as cables or semiconductor chips, used for data extraction or processing, are integrated into soft systems, localized stress concentrations can occur at the transition points between these rigid and soft materials. This, in turn, may lead to mechanical failure or damage at these critical junctions. These challenges have the potential to introduce unwanted noise and interference into the signal, thereby compromising the accuracy and consistency of the collected data [155]. Soft electronics employed in hand-motion recognition often necessitate real-time processing to facilitate seamless interactions with electronic devices or robotic systems. Unstable connections can introduce delays or interruptions in data transmission, adversely affecting the ability of systems to promptly respond to hand gestures. Strategies to mitigate this issue include the design of flexible interconnects or the incorporation of materials with intermediate mechanical properties to bridge the gap between rigidity and softness [159]. Furthermore, monolithic integration is considered an effective approach for amalgamating various electronic functionalities into a single, continuous structure [160]. Lastly, the development of chip-free wearable devices is being pursued as an alternative approach [161]. This approach obviates the necessity for discrete components, connectors, or rigid circuit boards, culminating in a more integrated and cohesive system.

### Conformity of the sensor on the skin in long-term usage

Hand-motion recognition through the utilization of soft sensors is predominantly conducted within controlled laboratory environments. Nevertheless, in real-world scenarios characterized by dynamism and harsh conditions, achieving consistent sensor performance necessitates meticulous consideration of adhesion and conformity to the skin. Factors such as the disparity in Young's modulus between the substrate and the skin, as well as the thickness of the substrate, exert a substantial influence. These variables can induce interference of signals and a failure in reflecting real hand motion, resulting from delamination of the soft sensor from the skin due to recurrent deformation. Efficient strategies to address these challenges involve the use of hydrogel-based devices designed to closely match the Young's modulus of the skin [162], or the adoption of ultrathin substrates (∼1 μm) to alleviate mechanical resistance [163]. Despite alterations in the mechanical properties of the substrate, wearable devices can still detach from the skin over prolonged periods due to sweat secretion. To tackle this challenge and ensure long-term stability in operation, research is focusing on the development of breathable electronics featuring porous substrates [164] and the creation of substrate-less biocompatible breathable nanomesh that can be directly applied to the skin [21].

### Hysteresis and non-linearity

The viscoelastic properties of soft materials used in soft sensors are characterized by their ability to exhibit both viscous (flow-like) and elastic (spring-like) behavior under mechanical stress [165]. This unique property allows them to deform and return to their original shape, but it also introduces certain challenges in hand-motion recognition. Hysteresis in soft materials occurs when their response to loading and unloading is not the same [155]. In the context of soft sensors, this means that when the material undergoes deformation due to hand motion, it may not revert to its initial state precisely and identically when the deformation is reversed. This hysteresis effect can lead to inaccuracies in the data collected from the sensors, impacting the precision of hand-motion recognition. In addition, soft materials often exhibit slow response times because of the time it takes for their viscoelastic properties to adjust to changes in mechanical stimuli. In dynamic hand-motion scenarios, rapid movements can challenge the ability of soft sensors to provide rapid real-time feedback. These delays in response time can hinder the ability to capture fast or sudden hand motions accurately. The behavior of soft materials can vary based on the rate at which they are deformed. Faster deformations may result in different responses compared to slower ones. Similarly, the response may differ based on the magnitude of deformation and the frequency of motion. This strain-rate dependency adds complexity to the behavior of the sensor, making it challenging to predict hand motions accurately across various speeds and degrees of movement. As a result of hysteresis and strain-rate dependency, the data generated by soft sensors can become inherently uncertain and non-linear. The relationship between the hand motion and the output is not straightforward, making it difficult to develop predictive models that can consistently and accurately interpret the data. While machine learning may be able to address some of these issues to a certain extent, fundamentally solving these problems requires a materials-oriented approach to polymer design to control the deformation of soft materials in a dynamic environment [166].

### Signal decoupling

Generally, mechanical sensors detect and respond to various mechanical stimuli such as strain and pressure, not just one target mechanical stimulus. However, in real-world applications, these sensors often face the challenge of ‘decoupling’, which means separating or distinguishing between different types of mechanical inputs to accurately determine the motion of the human hand. This decoupling issue becomes more pronounced when multiple mechanical stimuli are applied simultaneously, leading to signal overlap and confusion. One solution to the decoupling issue is the use of multimodal receptors. These receptors are designed to react differently depending on the types of mechanical stimuli they encounter. By having sensors with distinct response patterns to different inputs, it becomes possible to generate unique signal patterns for each type of mechanical stimulus [62,167]. These distinctive signal patterns can then be processed to separate and interpret the different inputs. Machine-learning techniques play a pivotal role in hand-motion recognition. Advanced machine-learning algorithms can be applied to analyze the sensor data generated by multimodal receptors [168]. These algorithms can focus on various features, such as slope, frequency and amplitude of the sensor signals, to differentiate between overlapping signals and precisely identify the specific hand motions being performed.

Furthermore, in hand-motion recognition, several hand joints can move omnidirectionally. This means that the hand can move freely in any direction, making it challenging to determine the exact motion and gesture using a single sensor. To overcome the challenges posed by omnidirectional hand movements, researchers have explored the use of multidimensional sensors and sensor patterns. Multidimensional sensors provide data from multiple angles or dimensions around the hand, enabling a more comprehensive understanding of hand motion [29]. Sensor patterns refer to specific shapes of sensor design or arrangements of sensors on the hand or in the environment that can respond differently, even in omnidirectional scenarios [169].

### Calibration for different hand sizes

Human anatomy varies significantly from one individual to another. Differences in hand sizes, joint flexibility and muscle strength can lead to variations in how a sensor responds to the same motion. Therefore, to achieve high precision in hand-motion recognition, it is essential to calibrate soft sensors individually for each user. This customization ensures that the sensor accurately captures and interprets the unique characteristics of the hand movements of a specific user. However, in numerous real-world applications, individuals with varying hand sizes need to use the same technology consistently for extended periods. As hand conditions or gestures change depending on users, maintaining the accuracy of hand-motion recognition becomes a challenge. This necessitates the development of adaptive calibration techniques that can continuously adjust sensor parameters in real time. Machine-learning algorithms are being employed to compensate for some of the challenges associated with calibration. These algorithms can adapt to individual users over time, learning their unique hand-motion patterns and adjusting sensor responses accordingly [170]. For example, transfer learning has the potential to address some of the challenges associated with sensor calibration in hand-motion recognition [170]. Transfer learning allows users to start with a pre-trained model that has already learned features from a large data set or a similar task. This can significantly reduce the initial calibration burden by providing a well-initialized model that is already attuned to general hand-motion patterns. Furthermore, users can fine-tune the pre-trained model using a smaller, user-specific data set [145]. This process, known as fine-tuning, helps the model adapt to the unique characteristics of hand movements of each user. While this approach may alleviate the need for frequent manual recalibration, it also introduces new complexities in model training and data collection. Furthermore, transfer learning can enable continuous learning and adaptation. As users interact with the system, the model can update itself based on new data, improving its accuracy over time. This continuous learning approach reduces the need for periodic, manual recalibration. However, it is important to note that while transfer learning can alleviate some of the challenges associated with sensor calibration, it may not be a one-size-fits-all solution. There are still nuances and complexities in hand-motion recognition that may require user-specific adjustments. In this respect, standardization of calibration procedures is an ongoing effort in the field of hand-motion recognition. Establishing common protocols and guidelines for sensor calibration can help ensure consistency and comparability across different systems and applications. This standardization could simplify the calibration process and enhance the interoperability of hand-motion recognition technologies.

### Motion parameters for accurate hand-motion description in real time

As mentioned earlier, hand-gesture-recognition technologies using soft sensors currently struggle to provide real-time, detailed descriptions of hand gestures. For instance, accurately estimating the bending angles of joints along each axis in a 3D space is a highly challenging task, even with well-calibrated sensors in a controlled environment. Due to the multiple DOFs created by the multiple joints in the fingers, wrist and elbow, each joint can assume numerous positions in 3D space. Moreover, for comprehensive and practical applications in real-world scenarios, it is crucial to quantitatively represent the states of all hand-movement elements, including fingers, wrists and joints. This representation should include dynamic parameters such as bending angle, angular velocity and angular acceleration for real-time measurement of hand motion, rather than solely predicting hand-motion types. As a possible solution for this, in terms of dynamic parameters for hand-motion recognition in real time, controllable and consistent hysteresis and strain-rate dependency of the sensor can be helpful for precise and detailed hand-motion recognition [171]. If the deformation of sensors in response to hand movements such as speed and magnitude of deformation consistently exhibits hysteresis or strain-rate dependency, it could potentially be leveraged to extract quantitative and dynamic information about hand motion in reverse.

### Recognizing specific hand motions in the natural environment

In practical scenarios, the task becomes considerably more complex when it comes to discerning a particular hand action from among the myriad daily hand movements. Recognizing hand gestures in a natural environment can be much more challenging than classifying predefined hand motions in constrained situations, making it even more difficult to detect very simple ‘tapping’ gestures. Moreover, the system should differentiate very similar hand actions even when the wearable sensor generates similar signals. In this context, discriminating between distinct actions, even if they yield the same signal, can be accomplished through various methods. One such approach involves considering the intention of the user, which may be inferred from contextual cues or input patterns [172,173]. For instance, a simple ‘tapping’ gesture can be discerned using the speed, direction or force applied during hand motion. This underscores the necessity for dynamic parameters in detailed hand-motion recognition and the identification of user intentions. Furthermore, maintaining a sense of continuity and context through the analysis of previous signals and actions can assist in distinguishing nuances in motion. By tracking the sequence of actions or considering the history of user interactions, it becomes possible to comprehend the specific context and meaning underlying seemingly similar signals, even in the absence of a rigid standard.

### Number of data sets for machine learning

Machine learning is a technology adept at discerning specific features from extensive and unattributed data sets, and in practice it is a data-based technology, thus the nuances of data quality and diversity are crucial. Achieving optimal outcomes in gesture recognition often hinges on the diversity of the data used for training machine-learning models. This diversity extends beyond merely capturing different gestures; it also encompasses factors like individual anatomical variations and cultural distinctions. As mentioned earlier, human anatomy varies from person to person, leading to subtle differences in the way individuals perform gestures. Having a data set that accounts for these anatomical variations can enhance the adaptability and accuracy of the model in real-world scenarios. However, collecting a substantial and diverse data set from real-world sensors is often a time-consuming and resource-intensive process [174]. This can lead to challenges related to data scarcity and the computational load required for training machine-learning models. Simulation can be a valuable tool for generating synthetic data that mimic real-world conditions [175]. Simulated data allow for the creation of diverse scenarios, which can supplement limited real sensor data. However, it is essential to ensure that the simulation accurately reflects the complexities of the actual environment. Meta-learning is an approach that focuses on training models to learn quickly from a limited amount of data, often referred to as few-shot learning [21]. By learning from a small set of examples, meta-learned models can adapt rapidly to new situations, reducing the data requirements for each specific task. Transfer learning leverages models that have been pre-trained on large data sets to jumpstart the training process for new tasks with limited data [176]. Models pre-trained on tasks related to gesture recognition or sensor data analysis can be fine-tuned on smaller, task-specific data sets to achieve better performance with less data.

### Overfitting

Overfitting, a common concern in machine learning, remains pertinent in the context of gesture recognition. While some models may excel at recognizing specific gestures in the training data, their broader applicability to novel gestures or unseen scenarios can be uncertain [177]. Therefore, strategies for improving model generalization should be explored. For example, techniques such as data augmentation, regularization and transfer learning can help models adapt to new gestures and situations. Gesture-recognition models can benefit from continuous learning and adaptation. Regular updates and fine-tuning based on user feedback and evolving real-world conditions can enhance their effectiveness over time. Cloud-based data sharing offers a promising solution to overcoming data limitations in hand-gesture recognition [178]. Researchers and organizations can collaborate by pooling their data sets and sharing them in an online-based system. This collective effort results in a more substantial and varied data set, which is advantageous for training robust machine-learning models.

### System configuration

In the pursuit of precision, it is logical that adding more sensors to the hand would enhance measurement accuracy. However, it is also true that as the number of sensors increases, the size of wearable devices will naturally expand. Even with highly integrated devices, the rise in the number of sensors can introduce challenges related to data volume and computational load, potentially leading to latency issues. Therefore, it is essential to consider these factors.

Additionally, the interaction between sensors and machine-learning models presents another noteworthy aspect for consideration. The effectiveness of gesture recognition may, to a certain degree, hinge upon the seamless interpretation of raw sensor data by these models. Given the inherent constraints of wearable devices, particularly their energy limitations, determining the most appropriate machine-learning approach can be a complex task. The decision to favor cloud-based solutions or to opt for edge computing or on-device methods such as TensorFlow Lite is not straightforward [179]. While cloud solutions may offer enhanced computational capabilities, it is imperative not to overlook potential issues related to latency and data privacy [180,181]. Conversely, on-device methods introduce a unique set of considerations, primarily centered around the computational constraints of the device itself [182]. In sum, the domain of real-time hand-gesture recognition remains a captivating field, replete with numerous avenues awaiting further exploration.

## CONCLUSION

In conclusion, we reported hand-gesture recognition and its applications through the integration of wearable soft sensors and machine-learning technologies. Starting with the materials, structures and driving mechanisms for the sensors, we have covered a wide range of topics relating to real-time analysis of sensor data and diverse machine-learning algorithms. A selection of research projects on hand-gesture recognition with the integration of wearable soft sensors and machine learning is summarized in Supplementary Table S2. The hand is one of the most complex parts of the human body, and if we can accurately recognize hand gestures and also understand the grasp state for various objects, it will greatly facilitate progress in other areas of the body.

To enhance cognitive capabilities, it is imperative to develop machine-learning algorithms that replicate the way biological individuals perceive and process data. Ideally, the integration of sensor technology and machine learning should not be approached as separate undertakings but as a comprehensive study that regards both soft sensors and machine learning as integral components of an intelligent system. In this regard, biomimetic perception has emerged as a rapidly evolving research area [183]. In reality, biological individuals possess the remarkable ability to process multiple sensory inputs simultaneously, rather than being confined to recognizing a single target signal. While previous biomimetic research endeavors have predominantly concentrated on replicating hardware aspects, such as improving sensor sensitivity and efficiency through structural design, there is an increasing acknowledgment of the necessity to pivot towards emulating the cognitive processes of biological individuals in the realm of perception.

In addition, research into the seamless integration of soft sensors with soft actuators has emerged as a promising avenue, particularly in advanced technologies like human augmentation. Soft actuators, known for their flexibility and adaptability, are increasingly being used to enhance human mobility and dexterity. However, to harness their full potential, it is crucial to develop feedback mechanisms that enable accurate and responsive control. This interdisciplinary approach has tremendous potential for enhancing human–machine interactions and revolutionizing fields such as healthcare, rehabilitation and human augmentation. With ongoing advancements and relentless future progress in the trinity of soft sensors, soft actuators and machine learning, we can expect these technologies to continue evolving and transforming various aspects of our lives, ultimately improving our well-being and enhancing capabilities in our daily lives.

## Supplementary Material

nwad298_Supplemental_File
