# Supplementary material for: Machine-learned wearable sensors for real-time hand-motion recognition: toward practical applications
Source: Natl Sci Rev. 2023 Nov 27;11(2):nwad298. doi: 10.1093/nsr/nwad298 (PMC10776364; doi:10.1093/nsr/nwad298)
Supplement: nwad298_Supplemental_File [file nwad298_supplemental_file.pdf]

Supplementary Data

Machine Learned Wearable Sensors for Real-time Hand Motion Recognition: Toward Practical Applications in Reality

Kyung Rok Pyun[1#], Kangkyu Kwon[1,2,3#], Myung Jin Yoo[1], Kyun Kyu Kim[4], Dohyeon Gong[5], Woon-Hong Yeo[2,6], Seungyong Han[5], Seung Hwan Ko[1,7*]*

[1] Department of Mechanical Engineering, Seoul National University, 1 Gwanak-ro, Gwanak-gu, Seoul 08826, Republic of Korea
[2] IEN Center for Human-Centric Interfaces and Engineering, Institute for Electronics and Nanotechnology, Georgia Institute of Technology, Atlanta, GA 30332, USA
[3] School of Electrical and Computer Engineering, Georgia Institute of Technology, Atlanta, GA 30332, USA
[4] Department of Chemical Engineering, Stanford University, Stanford, CA 94305, USA
[5] Department of Mechanical Engineering, Ajou University, 206 Worldcup-ro, Yeongtong-gu, Suwon-si, Gyeonggi-do 16499, Republic of Korea
[6] George W. Woodruff School of Mechanical Engineering, Georgia Institute of Technology, Atlanta, GA 30332, USA
[7] Institute of Advanced Machinery and Design (SNU-IAMD), Seoul National University, 1 Gwanak-ro, Gwanak-gu, Seoul 08826, Republic of Korea

# K.R. Pyun and K. Kwon equally contributed to this work.

* Corresponding authors

Prof. Seung Hwan Ko (Email: maxko@snu.ac.kr)

Prof. Seungyong Han (Email: sy84han@ajou.ac.kr)

Table S1. Features, Advantages, and Limitations of Representative Machine-Learning Techniques for Hand Motion Sensing with Wearable Soft Sensors.

ML Technique/ Algorithm		Application in Hand Motion Recognition	Advantages	Limitations
**Supervised Learning**	**SVM**	• Classifying predefined hand gestures based on sensor data and distinguishing between complex motion patterns.	• Efficient for linearly separable data and effective in high-dimensional spaces.	• Choosing an appropriate kernel can be challenging. Sensitive to noise and may overfit in cases of overlapping classes.
	**KNN**	• Classifying based on the proximity of feature vectors ('k' closest data points) in a multi-dimensional space.	• No assumptions about data; simple and intuitive to implement.	• Computationally intensive with large datasets. Sensitive to irrelevant or redundant features.
	**Random Forest**	• Identifying complex hand motion patterns from sensor readings. Classifying by aggregating decisions from multiple decision trees.	• Handles large datasets with higher dimensionality.<br>• Gives estimates of feature's importance.	• Model size can get very large. Potential for overfitting if trees are too deep.
**Unsupervised learning**	**k-means**	• Clustering hand motion data to find patterns or group similar gestures. Identifying common hand motion trajectories	• Relatively simple and fast. Suitable for large datasets.	• Assumes clusters to be spherical. Requires specifying the number of clusters.
	**PCA**	• Reducing the dimensionality of hand motion data while preserving variance. Transforming data to a new coordinate system.	• Reduces overfitting. Improves model efficiency.	• Doesn't cater to nonlinear relationships. Potential loss of information.
**Deep Learning**	**CNNs**	• Used to process image or video data from camera-based systems, identifying spatial features that correspond to specific gestures.	• Excels at handling high-dimensional data and are particularly adept at capturing spatial dependencies in image or visual data.<br>• Can learn intricate patterns from large datasets.	• Require a large amount of data for training and are computationally intensive, necessitating powerful hardware.<br>• May also be perceived as “black box” due to their complexity.
	**RNNs**	• Used for processing time-series data, capturing temporal dependencies in motion sensor data.	• Robust to spatial variations, able to detect local patterns, and efficient parameter sharing.	• Traditional RNNs struggle with learning long-term dependencies due to vanishing/exploding gradient problem.<br>• Training RNNs can be computationally intensive.
**Advanced Learning**	**Transfer Learning**	• Pre-trained models on a large-scale dataset can be fine-tuned on hand motion data, transferring learned features to the new task.	• Significantly reduce the amount of training data needed and speed up the learning process, as it leverages knowledge learned from related tasks.	• The effectiveness can be dependent on the similarity between the source and target tasks.
	**Adaptive Learning**	• Models can be updated or adapted over time to accommodate individual user’s styles and changes in their movements.	• Allow the model to keep improving and adapting to new, unseen data or changes over time, making it more robust to variations.	• Implementing needs a good strategy for adaptation.<br>• Care must be taken to prevent overfitting to recent data at the cost of forgetting older, yet

				still relevant, information.

Table 2. Summarization of research on the seamless integration of wearable soft sensors and machine-learning for hand posture detection.

Ref.[1]	Materials	Sensing Area	Data Modality	Sensing Mechanism	Sensitivity	Sensing Range (Stretchability)	Algorithms	Applications	Classification Accuracy
**[141]**	CNT	Finger	Strain	Piezoresistive	GF[2]: 0.82 (0 – 40% strain)<br>GF: 0.05 (60 – 200% strain)	0 – 280% strain	-	-	-
**[142]**	Ag NWs/PVDF-TrFE electrospun fibers	Wrist /Finger	Strain	Piezoresistive	GF: 5.326 (0 – 25% strain)	0 – 100% strain	-	-	-
**[145]**	EGaIn	Finger	Strain	Piezoresistive	GF: 2.493 (0 – 100% strain)	0 – 100% strain	-	Posture visualization	-
**[146]**	Silicone-Carbon black conductive layer/Silicone dielectric layer	Hand	Strain	Capacitive	-	-	CNN	Hand gesture recognition	-
**[147]**	Ag ink	Forearm	EMG	-	SNR[3]: ~ 50	-	Hyperdimensi on computing algorithm	Hand gesture recognition	92.87%
**[58]**	Ag NPs with serpentine design	Wrist	Strain	Resistive	GF: 3000 (0 – 1% strain)<br>GF: 300 (0 – 15% strain)	-	LSTM	Hand motion recognition	96.2%
**[85]**	Ag NWs with kirigami design	Forearm	EMG	-	SNR: 27.9	0 – 400% stretchability	-	Machine Control (Drone)	-
**[148]**	Cr/Au serpentine shaped electrode	Forearm	EMG	-	SNR: ~ 22.5	0 – 30% strain	LDA	Machine Control (Drone)	91.1%
**[17]**	Ag NWs/TPU electrospun fibers	Wrist	EMG	-	GF: 1.21 (at 50% strain)<br>GF: 1.66 (at 100% strain)<br>GF: 4.4 (at 200% strain)<br>GF: 24.2 (at 400% strain)	~ 600% strain	Xgboost	Machine Control (Car)	-
**[149]**	Graphene	Forearm	EMG	-	SNR: ~ 12	~ 60% stretchability	CNN	Machine Control (Robotic Hand)	98.5%
**[18]**	Graphene	Forearm	EMG	-	SNR: ~ 9.5	~ 60% stretchability	CNN	Machine Control (Car/Drone)	99 %

**[150]**	CNTs powder/Silica Gel	Forearm	Pressure	Triboelectric	Sensitivity:[4] 0.6 V kpa^{-1} (0.5 – 3 kPa)	~ 502% stretchability	LSTM	Machine Control (Robotic Hand)	98.31%
**[19]**	Polyester/PDMS	Finger	Strain	Triboelectric	GF: 2.47 (20 – 90% strain)	~ 90% stretchability	SVM	Sign Language Translation	98.63%
**[151]**	Hydrogel with CNT composite	Finger	Strain	Resistive	GF: 0.98 (0 – 300% strain)<br>GF: 2.19 (300 – 800% strain)<br>GF: 3.35 (800 – 1800% strain)<br>Sensitivity: 0.062 kPa^{-1} (1 – 5 kPa)<br>Sensitivity: 0.022 kPa^{-1} (5 – 9 kPa)<br>Sensitivity: 0.008 kPa^{-1} (9 – 15 kPa)	~ 4075% stretchability	Bagging	Hand writing recognition	82 - 97%
**[20]**	Skin/PDMS & PVDF	Wrist	Pressure	Triboelectric &Piezoelectric	Output voltage: ~ 80 mV at 5 N<br>Sensitivity: 0.012 V N^{-1}	-	LDA	Key Board	92.6%
**[21]**	Ag-Au NWs	Wrist /Finger	Strain	Piezoresistive	GF: 10 – 12 (at 10% strain)	-	Meta-learning	Virtual Key Board	85%
								Object Recognition	82.1%
**[152]**	Commercial conductive film	Whole Hand	Pressure	Piezoresistive	-	-	CNN	Object recognition	87%
							CNN	Hnad gesture recognition	89.4%
**[153]**	Pt layer (crack) on PDMS	Finger	Strain	Piezoresistive	GF: ~ 74 (0.025 – 5.4% strain)	-	Random forest	Object recognition	92.2%
							Gradient boosting classifier		87.2%
**[22]**	CNTs/TPE	Finger	Bending	Triboelectric	Sensitivity: 0.4 V N^{-1}	-	CNN	VR application	98.3%
								AR application	96.7%
**[23]**	Skin/Ecoflex	Finger	Pressure	Triboelectric	Output voltage: ~ 300 mV at 3.5 N	0.1 – 3.5 N	CNN	Object recognition<br>VR application	96.88%
							SVM		96.41%

[1]References: To avoid confusion with reference numbers in the main manuscript, the reference numbers in the supplementary data are identical to those in the main manuscript.

[2]GF: gauge factor, $(\Delta R/R_0/)/\varepsilon$, where ΔR is resistance change, R_0 is the initial resistance, and ε is strain.

[3]SNR: signal-to-noise ratio (SNR) for EMG sensor.

[4]Sensitivity: ΔV/Pressure or ΔV/Force for PENG or TENG-based sensor, where ΔV is voltage change.
